# The strong competitive role of 2n pollen in several polyploidy hybridizations in *Rosa hybrida*

**DOI:** 10.1186/s12870-019-1696-z

**Published:** 2019-04-04

**Authors:** Shu-min Gao, Mu-han Yang, Fan Zhang, Li-juan Fan, Yan Zhou

**Affiliations:** 1 National Engineering Laboratory for Tree Breeding, College of Biological Sciences and Biotechnology, Beijing Forestry University, No. 35 Tsinghua East Road, Haidian District, Beijing, 100083 China; 2 Beijing Key Laboratory of Greening Plants Breeding, Beijing Institute of Landscape Architecture, No.7 Huajiadi, Chaoyang District, Beijing, 100102 China

**Keywords:** *Rosa hybrida* L., 2n pollen generation, Strong competition, Hybridization, Ploidy inheritable, True hybrids identified

## Abstract

**Background:**

2n pollen play a strong competitive role in hybridization and breeding of multiploids in *Rosa hybrida*. The ploidy inheritable characteristic of ‘Orange Fire’ × ‘Old Blush’ were analyzed.

**Result:**

The results of the cytological observations indicated that 2n pollen developed from the defeated cytoplasmic division or nuclear division in the meiosis metaphase II of PMC (pollen mother cell) in ‘Old Blush’. The natural generation rate of the 2n pollen in ‘Old Blush’ (2x) was about 1.39 in percentage of all male gametes, whereas the tetraploids in the F1 offspring possessed a high rate, i.e., 44.00%. The temporal and spatial characteristics of ‘Old Blush’ pollen germination on the stigma and growth in pistil of ‘Orange Fire’ and ‘DEE’ were observed, and the results suggested that the germination rate of 2n pollen on the stigma was not superior to that of 1n pollen, but that the proportion of 2n pollen increased to 30.90 and 37.20%, respectively, while it traversed the stigma and entered into style. The callose plug in the 2n pollen tube was significantly thinner than that of 1n pollen tube. And each trait involved in our experiment probably is very important for F1 morphological phenotypes.

**Conclusion:**

We conclude that 2n pollen are involved in hybridization and have a competitive advantage while it traversed the stigma and entered into style. The callose plug in the 2n pollen tube was may have strongly influenced the competitive process in *R. hybrida*.

**Electronic supplementary material:**

The online version of this article (10.1186/s12870-019-1696-z) contains supplementary material, which is available to authorized users.

## Background

Polyploidization, an important mechanism for species formation, occurs widely in plants. Genome replication has occurred in species over evolutionary time [1]. Polyploidy may be derived via many pathways, such as in the rapid and efficient unreduced gametes by the crossing of parents producing polyploidy in their offspring. The formation of fertile polyploids does not only promote genetic and diversity, but also facilitates polyploid breeding. The relevance of polyploidy to speciation is itself still a topic of some contention. In many cases it is not clear to differentiate between cause and effect. For example, unreduced gametes are more often produced in cold environments [2, 3].

2n gametes are able to transmit parental heterozygosity traits to offspring in a large ratio. These gametes are also likely to possess certain special adaptations as a result of this heterozygosity, with a great degree of positive correlation with adaptability [4]. The conifers of Cheirolepidiaceae survived potential crisis from extinction because they produced 2n pollen in large proportions and contributed considerably to the generation of polyploidy during the late Triassic Era [5]. Actually, polyploidy is likely to have strong potential for adaptability, as observed in the hostile environments of high altitudes [6].

Unreduced gametes provide a basis for research in plant cytogenetics and breeding, and promote the development of polyploid genotypes [7]. In order to obtain polyploid plants, 2n gametes are preceded by chromosome doubling in mitosis during polyploid breeding. 2n gametes transfer genes with advanced traits from wild species into cultivars, transmit parental heterozygosity to their offspring, and improve inbreeding and pollen fertility in triploids [8]. Polyploid breeding is likely to confer significant economic and social benefits. Bilateral sexual polyploidization with the 2n gametes of *Medicago sativa* resulted in increased biomass, early flowering, large seed lots and weight, as well as big leaves with large cells [9]. Triploids with horticulturally important traits, such as vigorous growth and large sized flowers, were identified in hybrid offspring of tulip (*Tulipa gesneriana*) from crossing 2x × 2x plants hybrids [10], while tetraploids and pentaploids were found in the hybrid offspring of 3x × 2x hybrids [11]. Using 2n gametes, economically important traits including resistance to both abiotic and biotic stresses as well as quality-related traits like high specific gravity and slowly reducing sugars, were introgressed into cultivated potatoes from wild species [12]. Microsatellite genotyping indicated that most viable seeds of a parthenocarpic somatic variant were formed by the fertilization of unreduced gametes [13].

Most modern commercially valuable rose varieties are tetraploids, derived from long-standing interspecific hybridization between European tetraploids and Asian diploids [14]. It is clear that interspecific hybridization has played a key role in the evolution of the genus *Rosa*, but high levels of infertility or even abortion exist in triploid *Rosa* plants and interspecific hybrids [15]. Only eight to 11 wild species have contributed to modern commercially valuable rose breeding and selection [16], i.e., just a small fraction of the GenBank resources that could be used for genetic improvement. Interspecific hybridization is an important strategy for enriching genetic diversity and generating new germplasm resources [17]. Interspecific hybridization has great potential in improving both the number of traits as well as their quality, such as by increasing the size of the flowers, improving the flavor quality, changing the color, enhancing biological and abiotic stress resistance, as well as other characteristics [18, 19]. However, it remains unclear how the level of ploidy impacts sexual reproduction in *Rosa* plants. It is speculated that higher ploidy may lead to greater intra-species diversity in reproductive systems [20].

Two diploid Chinese rose varieties and six diploid rose varieties were used as the females for crossing with hexaploid rose varieties. Their offspring included plants with five types of ploidy, including diploids, triploids, tetraploids, pentaploids, and hexaploids [15]. In cytological observations during the meiosis stage, *Rosa* diploid and tetraploid varieties often exhibit perfectly matched chromosomes.

The pollen of tetraploids, hexaploids, and pentaploids can obtain one half of the paired chromosome segregation, and the eggs obtained one half of the paired chromosome segregation and unpaired univalence after meiosis in the *Rosa* family [21–24]. Some hybridized offsprings, such as *Rosa canina* and *Rosa inodora*, and their naturally occurring reciprocal hybrids, *Rosa dumalis* (5x) and *Rosa agrestis* (5x, 6x) are characterized by an asymmetrical meiosis enabling recombination in biparentally inherited chromosomes but preventing it in maternally inherited ones [25]. Apomixis may lead to higher ploidy levels in the offspring than in their parents, which only shown as a rather rare phenomenon in section *Caninae* [23], but does not occur in other roses including *R. hybrida.* In *R. alba* hybrids, 2n pollen was produced and abnormal ploidy was observed [14]. The pentaploid and hexaploid offspring were obtained when pentaploid *R. canina* varieties, as female parents, were crossed with modern tetraploid rose varieties as the male parents, and hexaploid offspring were produced due to the unreduced paternal pollen [26]. Higher ploidy levels in the offspring of rose hybridization experiments have been documented, and it is suggested that these may be caused by parental 2n pollen production or by self-pollination [27]. *Rosa hybrida* ‘HW336’, an interspecific diploid hybrid, originated from a cross between a dihaploid rose, *R. hybrida* ‘H190’. The diploid species *R. wichurana* produced 2n pollen in the ratio of 1.10~24.50% under various high temperature treatments and exhibited specific morphological variation in the amount of colpis and ectexine deposition, resulting in less striated structures as observed by scanning electron microscopy [28]. High temperatures led to increases in the number of 2n pollen from the diploid rose ‘HW154’, with the probability of dyads, triads, or polyads ranging from 0 to 6.20%, and 40 tetraploid hybrids among the 95 offsprings which generated from the crosses of diploid male parents with tetraploid female parents and analyzed by flow cytometry [29].

Based on the observation of PMC (pollen mother cell) meiosis in *R. hybrida*, *Ranunculus laetus*, *Agave tequilana,* etc., the presence of 2n pollen was confirmed by detecting the ploidy and heterozygosity of the hybrid offspring [30–32]. The degree of genetic heterozygosity from the parents of *R. wichuraiana*, *R. rugosa,* and two diploid roses and their hybrid offspring was detected using AFLP (amplified fragment length polymorphisms) in order to confirm the existence of the two types of 2n pollen: FDR (first division restitution) and SDR (second division restitution). 2n gametes produced by FDR transmit 80 to 100% of the parental heterozygosity to the progeny. In contrast, about 40% of the parental heterozygosity is expected to be transmitted by a SDR 2n gamete [4]. Researchers generally regard 1.3~1.5 times 1n pollen diameter as the critical value for identifying whether pollen is 2n pollen with diploid chromosomes [30–32]. 2n pollen with a diameter greater than 60 μm was observed in 55 dihaploid plants originating from the dihaploids of tetraploid roses, including ‘Anna’, ‘Leonidas’, ‘Sweet Promise’, and the parallel spindle was also observed to develop into an FDR dyad, while the vertical spindle developed into the FDR triad during meiosis of PMC [33]. There have been several reports of 2n pollen formation in Chinese roses at the cellular level, and the strong competitive advantage of 2n pollen has only been evaluated by examining the ploidy of the hybrid offspring. It is thus unclear how the Chinese rose produces 2n pollen. Furthermore, the identification of true hybrids is lacking, as is the cellular and molecular mechanisms of pollen competition in the pistil. It is uncertain whether this production is involved in the normal process of fertilization or apomixis, or whether the 2n male gametogeny and characteristics of its 2n pollen possess sufficient strength in the hybridization breeding of multiploids [34]. In our study, four hybrid combinations of tetraploid roses acting as female parents, and diploids acting as male parents, were investigated. We observed and authenticated the generation of 2n pollen in diploid male parents, the hybrid identification of the F1 offspring, the genetic characteristics of the ploidy, and the morphological traits of the hybridized combination of ‘Orange Fire’ × ‘Old Blush’. Furthermore, we used flow cytometry, SSR (simple sequence repeat) and SRAP (sequence-related amplified polymorphism) molecular marker identification techniques using the root-tip compression method, and observed the spatio-temporal growth characteristics of the pollen (‘Old Blush’) in the pistil (both ‘Orange Fire’, and ‘DEE’) after pollination. Our results elucidated the process of 2n pollen production in roses and its competitive advantages, and provide a basis for crossbreeding.

## Results

### The strong competitiveness of the 2n pollen in the four hybrid combinations

**Fig. 1:**
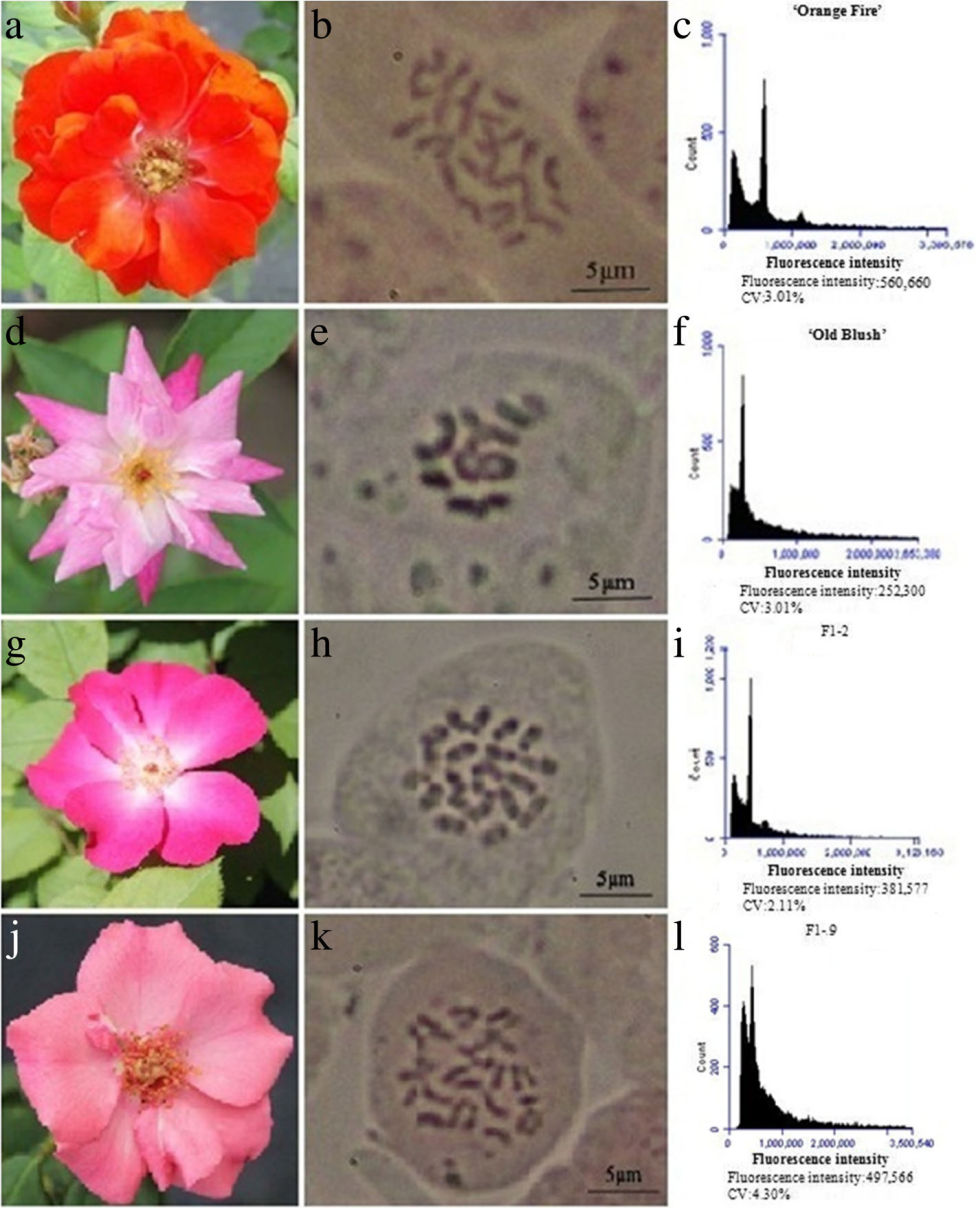
Genetics of ploidy characteristics of ‘Orange Fire’ × ‘Old Blush’. **a** ‘Orange Fire’. **b** Somatic cell chromosomes of ‘Orange Fire’. **c** Chromosome ploidy picture of ‘Orange Fire’ detected by flow cytometry with a fluorescence intensity of 560,660. **d** Flower of ‘Old Blush’. **e** Somatic cell chromosomes of ‘Old Blush’. **f** Chromosome ploidy picture of ‘Old Blush’ detected by flow cytometry with a fluorescence intensity of 252,300. **g** Flower of No.2 F1 (2n = 3x = 21) from ‘Orange Fire’ × ‘Old Blush’. **h** Somatic cell chromosomes of No.2 F1 (2n = 3x = 21) from ‘Orange Fire’ × ‘Old Blush’. **i** Chromosome ploidy picture of No.2 F1 from ‘Orange Fire’ × ‘Old Blush’ detected by flow cytometry with a fluorescence intensity of 381,577. **j** Flower of No.9 F1 (2n = 4x = 28) from ‘Orange Fire’ × ‘Old Blush’. **k** Somatic cell chromosomes of No.9 F1 (2n = 4x = 28) from ‘Orange Fire’ × ‘Old Blush’. **l** Chromosome ploidy picture of No.9 F1 from ‘Orange Fire’ × ‘Old Blush’ detected by flow cytometry and a fluorescence intensity of 497,566

The ploidy of the parents and F1 offspring in our four hybrid combinations was analysed by flow cytometry and compression method for the chromosomes of the stem tips, and showed a 100% precision rate in the ploidy identification (Table 1). The ratio of triploids and tetraploids in the F1 hybrid offspring of ‘Orange Fire’ (2n = 4x = 28, 1C = 0.79 pg, Fig. 1a, b, and c) × ‘Old Blush’ (2n = 2x = 14, 1C = 0.71 pg, Fig. 1d, f, and e) was 14:11, in which F1–2 (Fig. 1g, h and i) were triploid (2n = 3x = 21, 1C = 0.72 pg) and F1–9 (Fig. 1j, k and l) were tetraploid (2n = 4x = 28, 1C = 0.70 pg). In the F1 of ‘Chun Chao’ (2n = 4x = 28, 1C = 0.79 pg) × ‘Slater’s Crimson China’ (2n = 2x = 14, 1C = 0.71 pg) this ratio was 3:1; in the F1 hybrid offspring of ‘DEE’ (2n = 4x = 28, 1C = 0.73 pg) × ‘Slater’s Crimson China’ it was 5:1; and in the F1 hybrid offspring of the ‘DEE’ × ‘Old Blush’ it was 21:11. The somatic cell chromosomes and the chromosome ploidy of the stem tips of the F1 hybrid of ‘Orange Fire’ × ‘Old Blush’ is partly shown in Additional file 1: Figure S1. A ploidy diagram of the F1 hybrid of ‘Orange Fire’ × ‘Old Blush’ detected by flow cytometry is shown in Additional file 1: Figure S2, a ploidy diagram of the F1 hybrid of ‘Chun Chao’ × ‘Slater’s Crimson China’ detected by flow cytometry is shown in Additional file 1: Fig. S3, a ploidy diagram of the F1 hybrid of ‘DEE’ × ‘Slater’s Crimson China’ detected by flow cytometry is shown in Additional file 1: Fig. S4, and a ploidy diagram of the F1 hybrid of ‘DEE’ × ‘Old Blush’ also detected by flow cytometry is shown in Additional file 1: Figure S5. The DNA amounts of parents and F1 hybrids of ‘Orange Fire’ × ‘Old Blush’ detected by flow cytometry is shown in Additional file 2: Table S1, the DNA amounts of parents and F1 hybrids of ‘Chun Chao’ × ‘Slater’s Crimson China’ detected by flow cytometry is shown in Additional file 2: Table S2, the DNA amounts of F1 hybrids of ‘DEE’ × ‘Slater’s Crimson China’ detected by flow cytometry is shown in Additional file 2: Table S3, the DNA amounts of ‘DEE’ × ‘Old Blush’ detected by flow cytometry is shown in Additional file 2: Table S4.

**Table 1:** Ploidy traits of parents and F1 offspring in four hybrid combinations

Code	Parent ploidy		Number of F1 offspring		Per cent tetraploids
	tetraploid mother	diploid father	3x	4x	
1	‘Orange Fire’	‘Old Blush’	14	11	44.00%
2	‘Chun Chao’	‘Slater’s Crimson China’	24	8	25.00%
3	‘DEE’	‘Slater’s Crimson China’	25	5	16.67%
4	‘DEE’	‘Old Blush’	21	11	33.33%

### The natural rate of generation and viability of 2n pollen in ‘old blush’

**Fig. 2:**
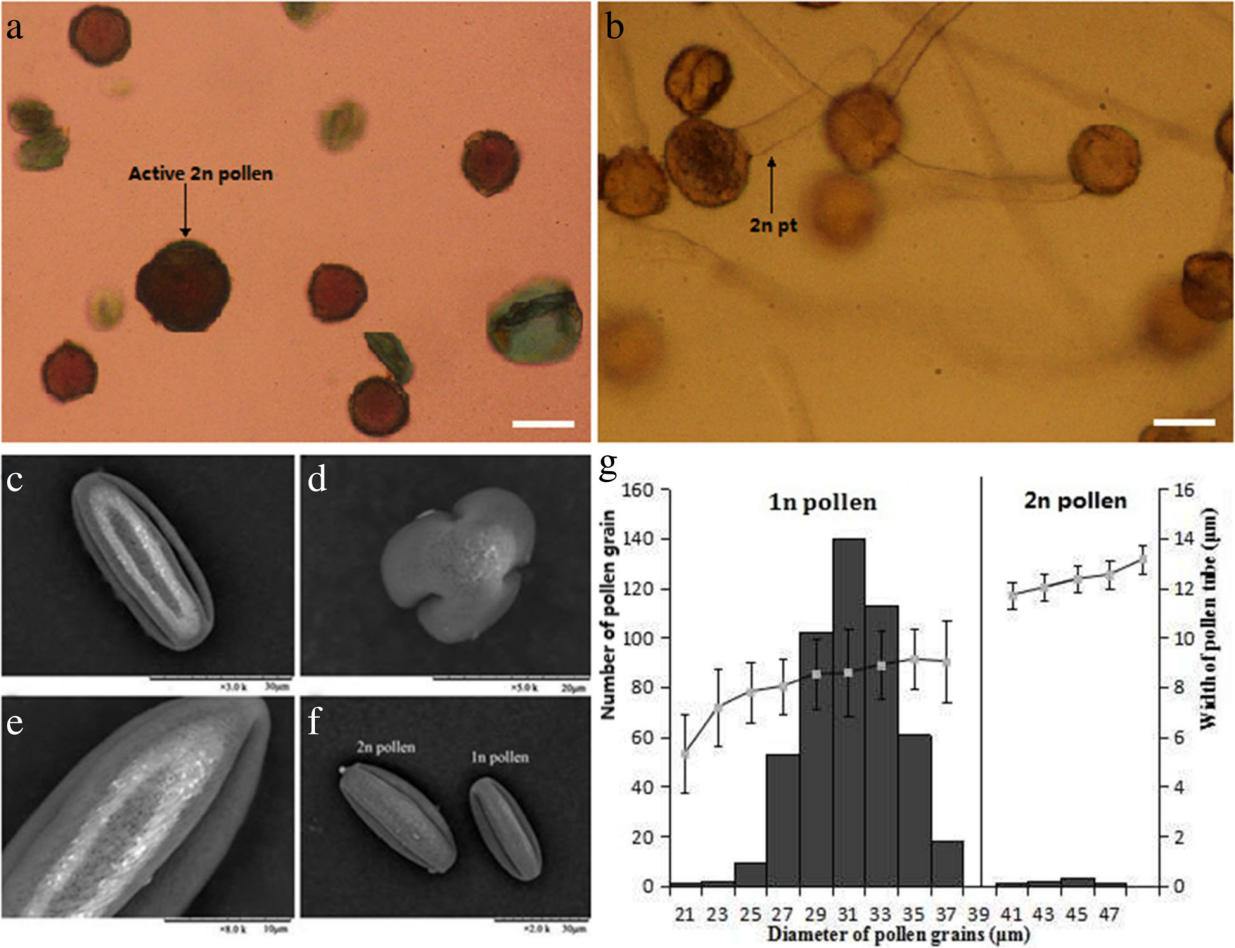
The morphological characteristics and germination of natural 1n pollen and 2n pollen of ‘Old Blush’. **a** Activity of natural 1n pollen and 2n pollen of ‘Old Blush’, stained by Alexandria dye, in which blue dye suggests pollen inactivity and red shows active generation. **b** In vitro germination of ‘Old Blush’ pollen. c-f. The morphological characteristics of pollen grains of ‘Old Blush’ under SEM. **c** Equatorial view. **d** Polar view. **e** Exine ornamentation. **f** 2n and 1n pollen. **g** Determination threshold of 1n and 2n of pollen grains diameter and pollen tube width. r (diameter, and width) = 0.97. bar A,B = 30 μm

The diameter of the natural 1n pollen and 2n pollen conformed to a normal distribution. The diameter of the 2n pollen was 44.20 ± 2.33 μm and was approximately more than 30% larger in size than the 1n pollen (Fig. 2a, g). The natural 2n pollen generation rate was about 1.39% in ‘Old Blush’ (Fig. 2a, g). The viability of the 2n pollen was about 25% as determined by Alexander dye staining (Fig. 2a). However, the proportion of tetraploids in the F1 hybrids was 44.00% (Table 1), which was much higher than the natural 2n pollen generation rate. This result may be due to the fact that 2n pollen has a certain competitive advantage during fertilization and development. The results indicate that 2n pollen is a strong competitor in the four hybrid combinations. Furthermore, the pollen tube width and pollen grain diameter exhibited a significant positive correlation (Pearson correlation coefficient r = 0.97), and the 2n pollen tube width was more than 1.3 times as wide as the 1n pollen (Fig. 2b, g), which could be used to determine a 2n pollen tube in the pistil.

The pollen of ‘Old Blush’ was observed as perprolate in shape under the scanning electron microscope, with longitudinal and parallel extine sculptures, wide and flat ridge, and perforated ridge ditch depths (Fig. 2c-f).

### Cytological observations of 2n pollen generated in the ‘old blush’ diploid

**Fig. 3:**
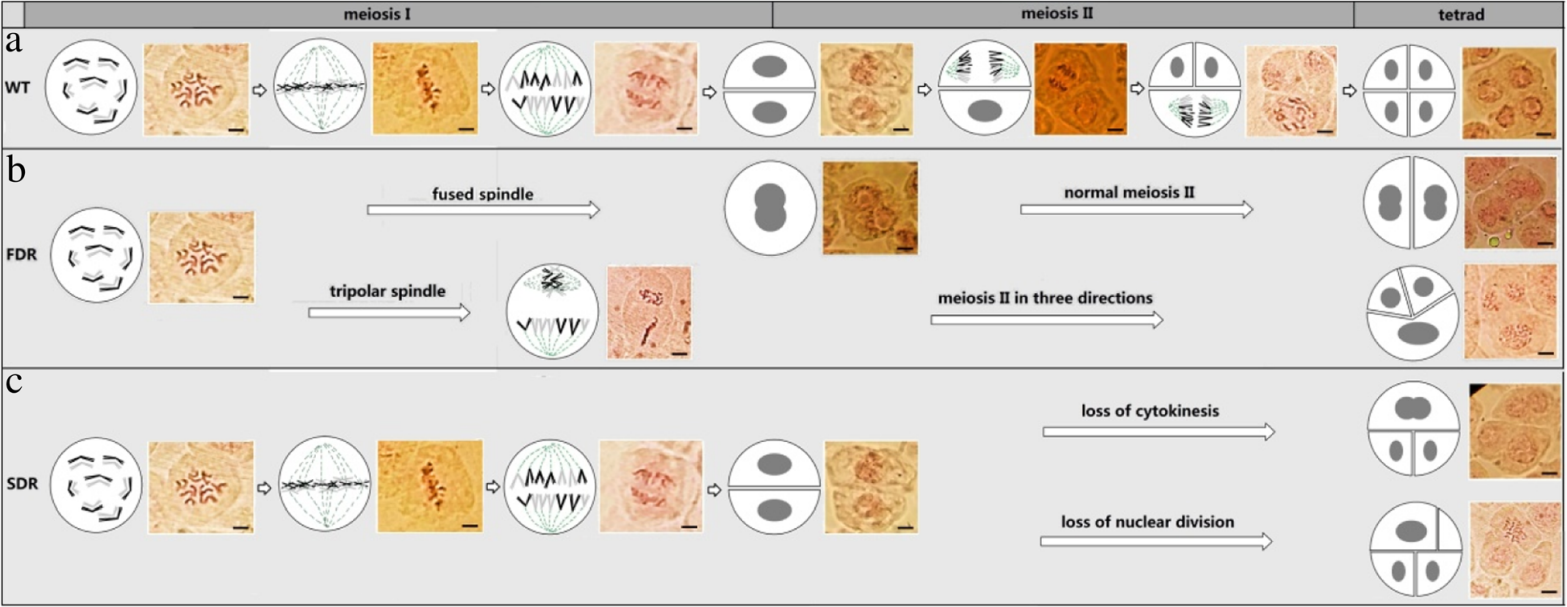
Different pathways of natural 2n pollen production. **a** Meiosis of normal PMC. After synapsis and separation of the homologous chromosome, PMC developed into a dyad of which chromosome disappeared and nucleus became visible. Chromosome in each cell of dyad had reappeared and then terads were formed after meiosis II. Meiosis in ‘Old Blush’ is the successive type and is asynchronous. **b**. production process of two types of FDR 2n pollen. One process is PMC with fused spindles that develop into an FDR dyad formed of two cells with a fused nucleus after normal meiosis II, while another process is the development of the vertical spindle into an FDR triad with two 1n cells and one 2n cell from the triple spindle. **c**. two production pathway of SDR 2n pollen. A dyad formed after normal meiosis I, and then developed into an SDR triad with one binucleate cell and two 1n cells formed from the loss of cytoplasmic division, or abnormal nuclear division caused by the formation of an SDR tetrad with one 2n cell, two 1n cells, and one clear cell

Given that we obtained the F1 tetraploid offspring of ‘Orange Fire’ (4x) × ‘Old Blush’ (2x), we speculated that the unreduced 2n pollen was generated in the diploid ‘Old Blush’ and was involved in fertilization. A few anomalies in the meiosis process were found, while the entire process of cell division was basically normal, including two divisions of both the cell nucleus and cytoplasm as successive types. At the prophase I stage, the nuclear chromosomes replicated completely and the chromatin gradually turned to contraction and homologous chromosome pairing occurred at the zygotene stage. Sibling chromatid fragments were exchanged at the diplotene stage. At the metaphase I stage, chromosomes lined up on both sides of the equatorial plate, reaching their shortest length, and became distinctly discernible in bivalent modality. At the anaphase I, the homologous chromosomes separated, moving to the poles under the draw of the spindle fiber, while at the telophase I the chromosomes gradually moved closer to the poles. The chromosomes began to break the spiral, returning to the chromatin state. At prophase II, the chromatin gradually became concentrated, and spiraled to a form of seven chromosomes with sibling chromatids. At metaphase II, the chromosomes were arranged sequentially on the equatorial plate, while the vertical spindles could be observed under normal conditions. In the anaphase, each chromosome centromere separated, while two sibling chromatids separated as well, resulting in two chromosomes that exhibited dipole movement under the guidance of the spindle fibers. In telophase II, the chromosomes reached their fixed positions under the guidance of the spindle fibers, while the chromosomes gradually broke the spiral, returning to the chromatin state with the reformation of the nucleolus. Most cells became tetrads after cytoplasmic division, including a plane-shaped tetrad, a tetrahedral tetrad, or a line-shaped tetrad (Fig. 3a).

In comparison with normal meiosis, ‘Old Blush’ can naturally generate two types of 2n pollen: FDR and SDR. The FDR 2n pollen was developed from both the fused spindle and the vertical spindle in two ways; namely, the former FDR dyad with two fused nuclei after meiosis II as a result of the failed separation of two nuclei during meiosis I stage, the latter eventually forming an FDR-type triad composed of two 1n-cells and one 2n-cell, which developed from the first meiosis of the late meiosis I spindle to the tertiary spindle (Fig. 3b). The SDR 2n pollen was developed from two types of abnormal division taking place after normal meiotic I. One was an SDR triad with a 2n fusion nuclear cell and two 1n cells, which is due to the lack of cytokinesis. The other was the SDR tetrad with a 2n cell, two 1n cells, and one vacancy cell due to a lack of nuclear division (Fig. 3c). The FDR dyad (0.60%) produced two 2n pollen, the FDR triad (0.80%), SDR triad, or tetrad (total of 1.60%) each produced 2n pollen, ultimately producing 0.51% FDR 2n pollen and 0.41% SDR 2n pollen.

### 2n gametes involved in F1 tetraploid generation by SSR and SRAP identification

**Fig. 4:**
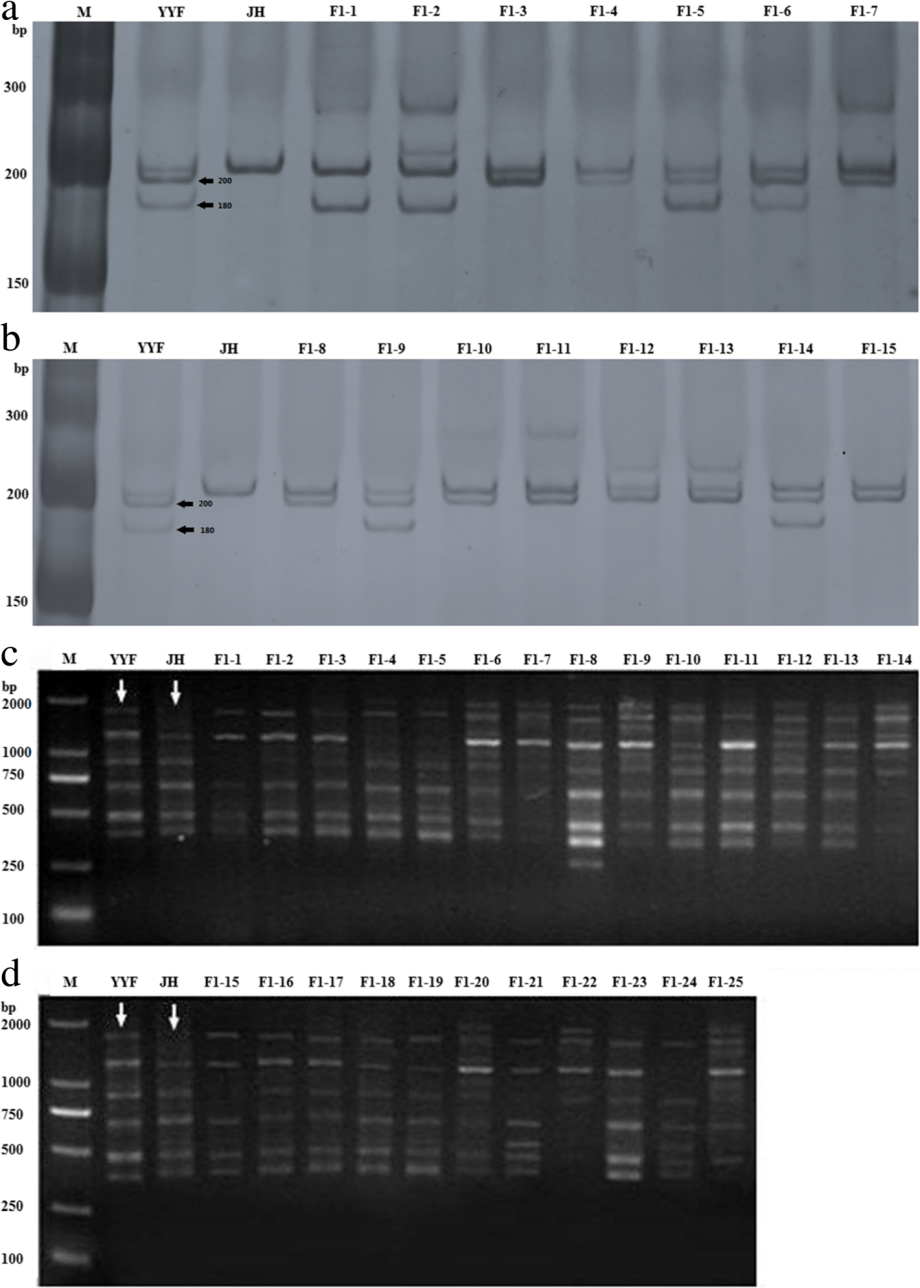
Results of SSR and SRAP for the F1 of ‘Orange Fire’ × ‘Old Blush’. Note: YYF, ‘Old Blush’; JH, ‘Orange Fire’; a. F1(1–7) from ‘Orange Fire’ × ‘Old Blush’.b.F1(8–15) from ‘Orange Fire’ × ‘Old Blush’. c. F1(1–14) from ‘Orange Fire’ × ‘Old Blush’. d. F1(15–25) from ‘Orange Fire’ × ‘Old Blush’. a, b. The black arrows indicate the location where the specific band of a male parent appeared. c, d. The white arrows indicate the location where the specific band of the parents appeared

The RW55C6 primers were amplified in the parents and the specific parental bands and high reproducibility of the amplification products was obtained with two target band sizes of about 200 bp and 180 bp (Fig. 4a, b). The primers in the offspring can also appear as good polymorphic bands. If the offspring has specific bands of both paternal and female parent, then we consider that the offspring is heterozygous. It is normal for both the 180 and 200 bp bands of paternal ‘Old Blush’ independently or simultaneously inherited to F1 hybrids based on the law of segregation and the law of independent assortment. The 29 hybrids detected using this primer in ‘Orange Fire’ × ‘Old Blush’ constitute true hybrids.

At 77.78%, the ripening rate of the hybrid combination ‘Orange Fire’ × ‘Old Blush’ was high, while the seed germination rate was relatively low at 35.71%, ending up with 25 F1 plants. We tested whether the F1 plants were true hybrids using SSR and SRAP molecular markers.

For SRAP, the final selected primer combinations were the following: for Me9: TGAGTCCAAACCGGGCT, and for empire 5: GACTGCGTACGAATTGTA, which were the most appropriate for amplification. The SRAP molecular markers of the F1 plants of ‘Orange Fire’ × ‘Old Blush’ are shown in Fig. 4c, d. The arrows in the figure point to the band that only occurs in the male parents. We chose this stripe as a specific stripe, which appeared in each F1 plant (no arrows in Fig. 4c, d). It implies that all F1 plants are probably true hybrids of the male and female parents, ruling out inbreeding by incomplete castration and concluding that the 2n gametes probably originated from the male parents in the F1 tetraploid.

### The germination and growth of 2n pollen in ‘old blush’ and fructification characters in the pistil of ‘Orange fire’ and ‘DEE’

**Fig. 5:**
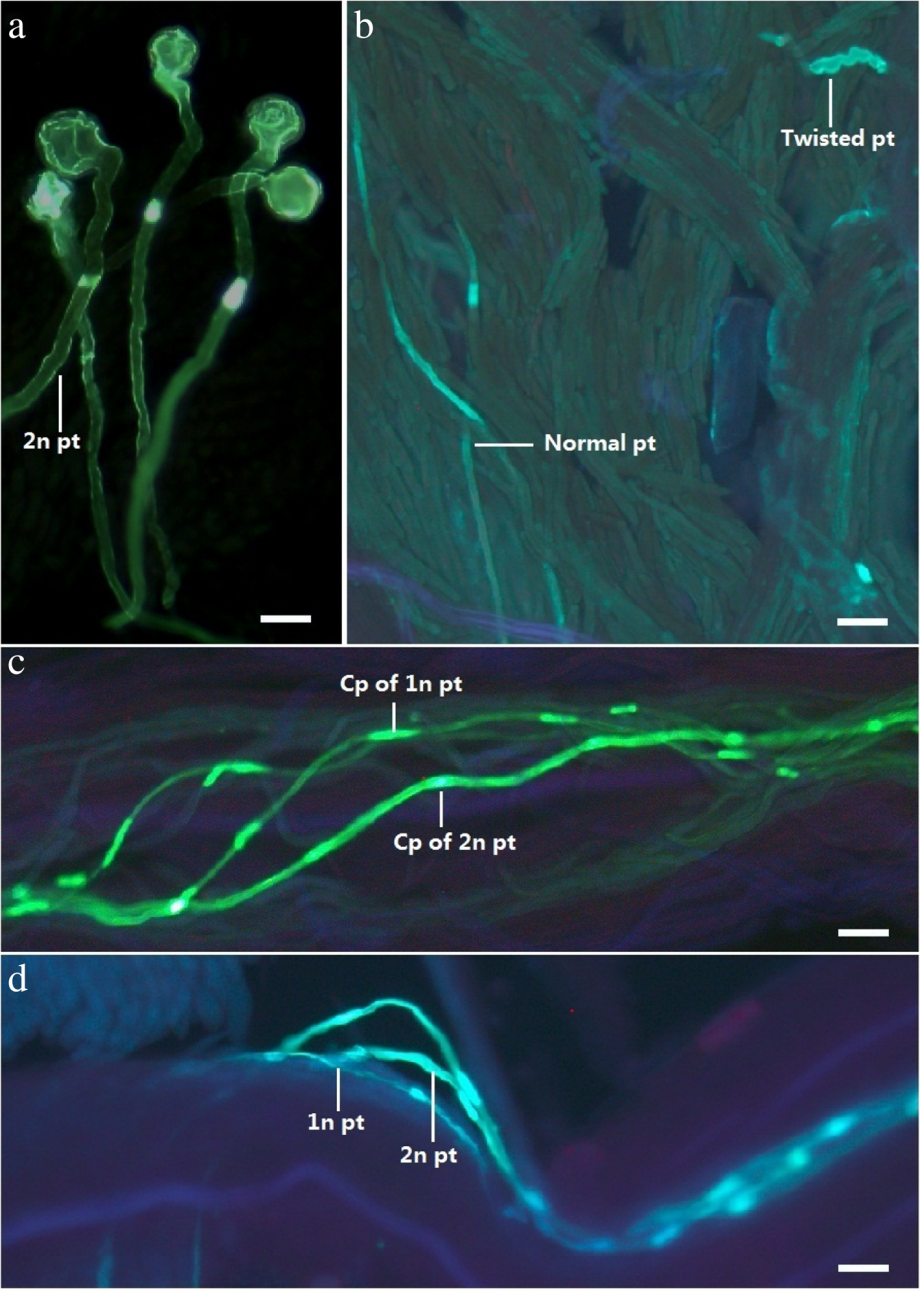
Germination of 1n and 2n pollen on pistil of ‘Orange Fire’. **a** ‘Old Blush’ pollen germinated on the surface of ‘Orange Fire’ stigma. **b** Pollen tubes in the transmitting tissue at the junction of stigma and style where shows the strongest intensity of pollen competition. The part of the pollen tube is twisted at the apical region and stops growing while normal pollen tubes grow through the stigma into the style. **c** Pollen tubes in the style at 48 h after pollination. d. Pollen tubes enter ovary at 72 h after pollination. pt.: pollen tube, cp: callose plug. A bar =25 μm, B~D bar = 40 μm

**Fig. 6:**
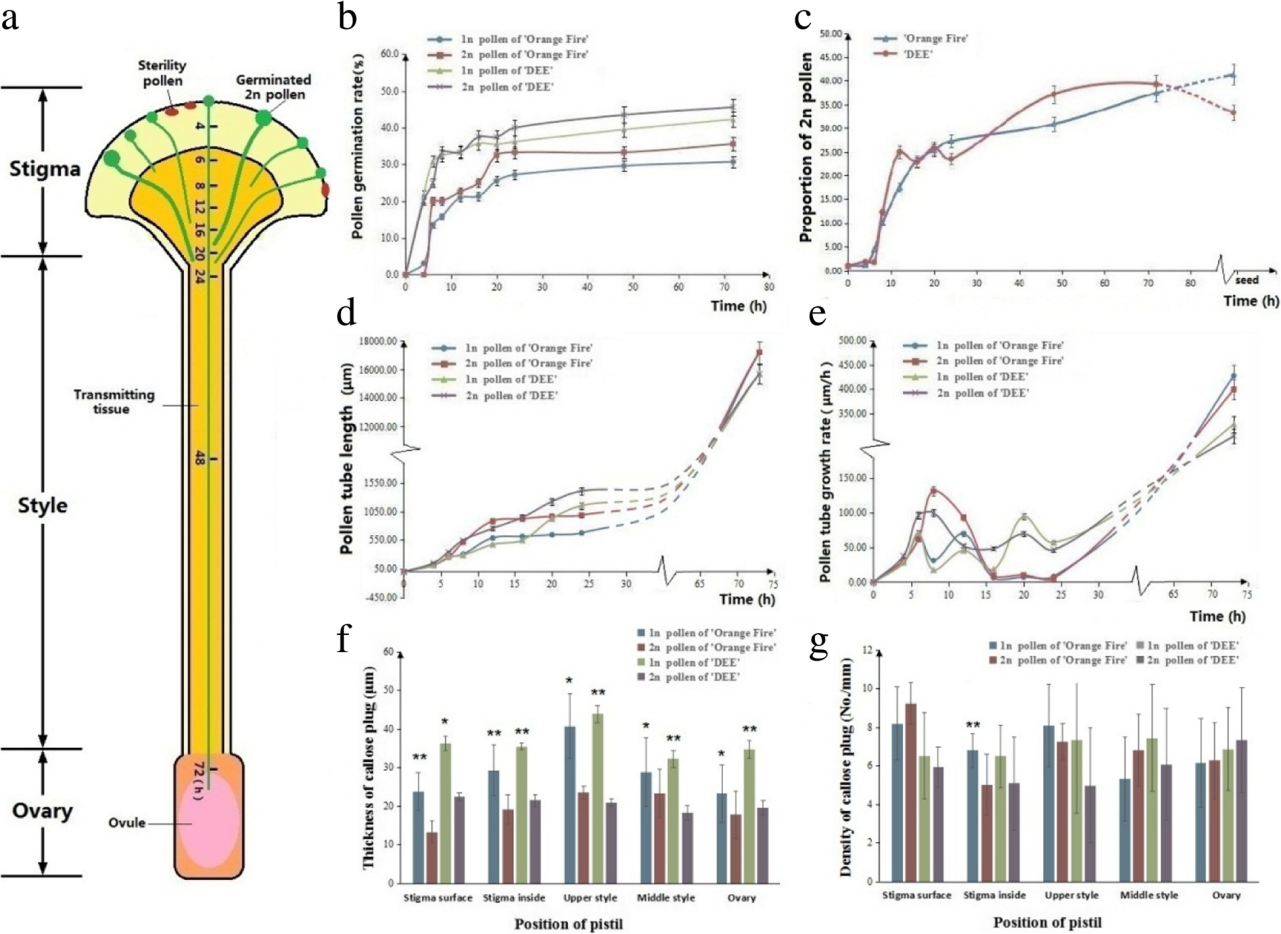
Progression of the pollen tube of ‘Old Blush’ growing in the pistil of ‘Orange Fire’ and ‘DEE’. **a** Progression in the time and position of ‘Old Blush’ pollen growing in the pistils. **b** The pollen germination rate varied with the hours after pollination. **c** The growth rate proportion of the 2n pollen tube per hour after pollination. **d** The rate of growth in length of the pollen tube per hour after pollination. **e** Pollen tube growth rate per hour after pollination. **f** The thickness of the callose plug at different positions in the pistil after the pollen tube entered the ovary (72 h). **g** The density of the callose plug at different positions in the pistil after the pollen tube entered the ovary (72 h)

The pollen in ‘Old Blush’ germinated on the stigmatic surface of ‘Orange Fire’ and ‘DEE’ after about 4 h of pollination (Fig. 5a, 6a). The germination rate predominantly increased to 24.00 ± 7.20% at 4–6 h, and then proceeded to increase slowly and was stable in 27.60 ± 4.80% of ‘Orange Fire’ and 39.70 ± 4.80% of ‘DEE’ 12 h later (Fig. 6b). There was no significant difference in the germination rate between the 1n and 2n pollen, as well as both ‘Old Blush’ and ‘DEE’ female parents. This indicated that the competitive advantage of the 2n pollen had not occurred during the pollen germination period. The ‘Old Blush’ pollen tube began to enter the internal superficial layer of the stigma and transmitted the tissue of ‘Orange Fire’ 6 h later (Fig. 5b, 6a), and successfully entered into the style 24 h later (Fig. 5c, 6a). The pollen tube exhibited an accelerated growth rate in the style and finally entered into the ovary 72 h later (Fig. 5d, 6a). Compared with the natural generation rate of 2n pollen (1.39%), the proportion of 2n pollen on the stigma of both the ‘Orange Fire’ and ‘DEE’ female parents was 4.50 and 1.73%, respectively (Fig. 6c). The proportion of 2n pollen in the internal superficial layer transmitting tissue increased significantly at 8 h by 10.00 and 12.24% respectively, and the rate of increase increased by 122.20 and 607.50%, respectively. The proportion of 2n pollen in the stigma internal deep layer transmitting tissue was 25.45 and 23.53%, respectively, at 20 h, and the rate of increase of ‘Orange Fire’ continually increased to 154.50%, whereas this rate decreased in ‘DEE’ by 92.20%. After entering the style (24 h), the proportion of 2n pollen gradually increased by about 30.90 and 37.20%, respectively, and the rate of increase was 21.40 and 58.10%, respectively. The rate of increase of 2n pollen tubes in the ovary still increased by about 33.90%, which led us to speculate about the 44.00% tetraploid F1 offspring of ‘Orange Fire’ (Fig. 6c). The above results indicated that the greatest rate of increase in the proportion of 2n pollen in ‘Orange Fire’ occurred in the internal deep layer transmitting tissue in the stigma (20 h), secondly in the internal superficial layer transmitting tissue of the stigma (8 h), and finally in the ovary (48 h). The internal deep layer thus exhibits strong competitive advantage. Conversely, the greatest rate of increase in 2n pollen in ‘DEE’ occurred in the superficial layer transmitting tissue (8 h), followed by internal deep layer transmitting tissue (20 h), and then the style (24 h). Thus the internal superficial layer exhibited a strong competitive advantage.

The length of the 2n pollen tube was longer than that of 1n pollen tube by about 300 μm after 8–24 h of pollination, which is during the stage when the transmitting tissue enters the style. The growth rate showed an obvious competitive relationship at this stage in both the female parents (Fig. 6d). Before entering the style, the 2n pollen tube grew fastest at 8 h at a speed of 100.06 μm/h and 131.42 μm/h, respectively, in ‘Orange Fire’ and ‘DEE’, during which the pollen growth rate of 1n was the slowest at 17.69 μm/h and 31.47 μm/h, respectively (Fig. 6e). The corresponding morphological features were observed at this point when the pollen tube grew inside the transmitting tissue. Distortion of the apical region of the pollen tube was observed in the connection area of the stigma and style, as well as pollen growth cessation caused by callose accumulation (Fig. 5c). After entering the style, the pollen tube growth rate significantly increased to 100 ~ 400 μm/h, reaching the ovary after 72 h (Fig. 6d). The competitive advantage of 2n pollen in both female parents was indicated by the increase in the proportion of 2n pollen inside the stigma leading to the upper part of the style (8–24 h after pollination). The proportion of increase in the 2n pollen tube was steady in ‘Orange Fire’ and constituted “once-off competitive type” (stigma internal deep layer strong competitive model). Conversely, the proportion of 2n pollen tube within the ‘DEE’ stigma fluctuated, and increased inside the style and then decreased before fertilization, which was indicative of the “two-off competition type” (stigma internal superficial layer strong competitive model).

To investigate the distribution characteristics of the callose plug in the pollen tube with different ploidy in vivo, the thickness and density of the callose plug at different areas of the pistils after the pollen tube entered the ovary (72 h) were measured (Fig. 6f, g). The callose plug in 2n pollen tube was highly significantly thinner than that of the 1n pollen tube in every area of the pistil in both female parents (Fig. 6f). The callose plug density in the 2n pollen tube was significantly lower than in the 1n pollen tube in the stigma of ‘Orange Fire’, while the callose plug density in the 2n pollen tube was lower than in the 1n pollen tube before reaching the ovary of ‘DEE’. The other pollen tubes with different ploidy did not exhibit any significant differences in the different areas of the pistils after the pollen tube entered the ovary (72 h). In combination with the observed pollen competition between the different ploidy in two female parents, the thickness and density characteristics of the callose plugs were determined to be closely associated with the strongly competitive 2n pollen.

### Principal component and correlation analysis of the phenotypic traits in F1 plants of ‘Orange Fire’ × ‘old blush’

The phenotypic traits of the F1 plants of ‘Orange Fire’ × ‘Old Blush’ were investigated and are shown in Table 2. The results of the principal component analysis of the phenotypic traits are presented in Tables 3 and 4. The main components contributing to the variance were relatively dispersed and the cumulative contribution increased slowly until the 5th main component, where the cumulative contribution reached 84.53%. In the analysis, three main components were extracted with characteristic roots greater than 1, accounting for 63.07% of the total amount of variation. The contribution of the first component was 29.39%, with a greater load capacity in top leaflet length, width, and the ploidy than the other traits, with absolute values greater than or equal to 0.70. The first principal component suggests that leaflet size is related to the ploidy of the F1 plants.

**Table 2:** Morphological and ploidy characteristics of the F1 of ‘Orange Fire’ × ‘Old Blush’

F1	Ploidy	Plant type	Color	Petal	Flower diameter(cm)	Top leaflet length (cm)	Top leaflet wide (cm)	Leaflet shape	Aculeus
F1–1	3x	bush	pinkish orange	polyphyll	5.60	4.05	2.32	ovoid	stingless
F1–2	3x	vine	pink	Single petal haplopetalous	5.25	4.90	2.97	ovoid	stingless
F1–3	3x	bush	red	Single petal	4.66	3.72	2.12	ovoid	small and dense
F1–4	3x	vine	red	Single petal	5.80	4.25	2.46	ovoid	different sizes and dense
F1–5	4x	bush	orange	Single petal	6.25	3.85	2.55	ovoid	small and sparse
F1–6	3x	bush	red	Single petal	5.90	4.18	1.98	lanceolate	small and dense
F1–7	4x	bush	pinkish orange	polyphyll	8.40	4.15	2.28	ovoid	big and sparse
F1–8	4x	vine	pinkred	Single petal	12.50	5.09	3.80	ovoid	big and fewer
F1–9	4x	vine	pink	Single petal	8.66	4.91	3.20	ovoid	small and dense
F1–10	3x	vine	red	Single petal	5.82	4.40	2.20	lanceolate	small and dense
F1–11	4x	bush	red	Single petal	6.45	4.95	3.45	ovoid	big and fewer
F1–12	4x	vine	red	Single petal	7.70	5.75	3.65	ovoid	different sizes and dense
F1–13	4x	vine	orange	Single petal	5.70	4.04	2.55	ovoid	small and sparse
F1–14	4x	vine	orange	semidouble	6.70	3.48	2.16	ovoid	big and fewer
F1–15	3x	vine	pink	Single petal	6.80	4.92	2.45	lanceolate	small and dense
F1–16	3x	bush	dark pink	Single petal	9.10	4.93	2.51	lanceolate	big and sparse
F1–17	3x	vine	pink	Single petal	6.10	4.05	2.24	ovoid	small and sparse
F1–18	3x	vine	pink	Single petal	7.90	3.70	1.82	lanceolate	small and sparse
F1–19	3x	vine	red	Single petal	4.90	4.97	3.10	ovoid	different sizes and dense
F1–20	3x	bush	red	Single petal	7.16	4.30	2.68	ovoid	small and sparse
F1–21	3x	bush	pinkred	Single petal	6.50	4.55	2.37	ovoid	Stingless
F1–22	3x	vine	red	Single petal	6.15	4.43	2.75	ovoid	small and dense
F1–23	4x	bush	orange	Single petal	8.20	4.14	2.72	ovoid	small and sparse
F1–24	4x	bush	orange	Single petal	8.50	5.10	3.53	ovoid	small and sparse
F1–25	4x	vine	pinkred	semidouble	7.60	5.85	3.05	ovoid	small and dense

Note: Plant type: small bush < 80 cm, medium and large bush 80–150 cm, vine > 150 cm; Petals: Single petals< 10, semidouble 10–20, polyphyll 20–60

**Table 3:** Component eigenvalues, contribution and accumulated contribution by components

Component	Eigenvalue	Concribution(%)	Accumulated contribution(%)
1	2.64	29.39	29.39
2	1.60	17.73	47.12
3	1.44	15.95	63.07
4	0.97	10.74	73.81
5	0.96	10.72	84.53
6	0.69	7.67	92.20
7	0.32	3.56	95.76
8	0.26	2.84	98.60
9	0.13	1.40	100.00

**Table 4:** Nine characteristic loadings on principal component axes

Characteristic	Component 1	Component 2	Component 3
Ploidy	0.70	0.43	0.09
Plant type	0.14	−0.51	0.36
Color	0.29	− 0.31	− 0.78
Petal	− 0.04	0.70	0.12
Flower diameter(cm)	0.51	0.14	0.62
Top leaflet length (cm)	0.72	−0.30	0.26
Top leaflet wide (cm)	0.88	0.01	−0.18
Leaflet shape	0.50	0.50	−0.42
Aculeus	0.50	−0.46	−0.05

The second principal component explained 17.73% of the variation, with larger loadings in the petals, plant type, and leaflet shape, with absolute values greater than or equal to 0.50. This component mainly reflected the characteristics of the flowers, leaves, and plants on the specific form of the F1 plants. The contribution of the third component was 15.95%, with larger loadings in color and flower diameter, mainly reflecting the ornamental features of the flowers of the F1 plants.

The three main component traits do not overlap and present high loading scores in which no single trait exerts a higher load on the three main components. This suggests that we cannot confirm any particular trait as the main characteristic of the F1 plant phenotype in this generation. Each of the phenotypic traits involved in the generation played a role in F1 morphogenesis and require a comprehensive description and evaluation.

Ploidy was significantly positively correlated with flower diameter, top leaflet width, and leaflet shape, which in turn were significantly and positively correlated with leaflet length and leaflet shape in the F1 plants of ‘Orange Fire’ × ‘Old Blush’ (Table 5). Ploidy is thus positively correlated with the size of both the flowers and leaves, i.e., the higher ploidy F1 plants have larger flowers and leaves, which is consistent with the theory that particular organs of high-ploidy plant varieties are larger than in low-ploidy plants of the same species as a result of the greater number of chromosomes in high-ploidy varieties [10, 35].

**Table 5:** Correlation matrix of nine phenotypic traits in F1 plants of ‘Orange Fire’ × ‘Old Blush’

	Ploidy	Plant shape	Color	petal	Flower diameter	Top leaflet length	Top leaflet wide	Leaflet shape	Aculeus
ploidy	1	−0.03	0.07	0.19	0.49*	0.23	0.47*	0.44*	0.19
plant shape		1	−0.15	−0.19	0.00	0.22	0.00	−0.04	0.28
color			1	−0.25	−0.22	0.03	0.28	0.17	0.38
petal				1	0.02	−0.15	−0.10	0.21	−0.03
flower diameter					1	0.35	0.26	−0.04	0.19
top leaflet length						1	0.69**	0.07	0.30
top leaflet wide							1	0.53**	0.28
leaflet shape								1	−0.03
aculeus									1

Note: *: The correlation was significant. **: The correlation was extremely significant

In addition to the earlier mentioned correlations between the traits, Table 5 shows other correlations, none of which were statistically significant. Every trait or characteristic selected for the trial were quite independent and could not be replaced by another trait. We might in the future consider increasing the ploidy of the offspring for the selective breeding of new varieties with larger flowers.

## Discussion

### Production of 2n pollen in *R. hybrida*

The cytological mechanisms of 2n pollen production in Chinese rose were revealed in this study. However, no molecular mechanisms or related genes were reported, thus warranting further investigation. In our study, in normal meiotic cells, there were two spindle apparatus with the spindles at 90° to each other present during metaphase II of the ‘Old Blush’ daughter cells in *R. hybrida.* However, in abnormal, undivided cells, the two spindles were parallel to each other or resembled a three-pole spindle apparatus when the two chromosome complements moved to the same area of the cell and formed dyads and triads during anaphase II. We thus proposed that 2n pollen was mainly generated from the two abnormal division processes in rose, including the fusion spindle and the triple spindle developed from the vertical spindle, as well as a loss of cytoplasmic division or abnormal nuclear division. During the process of meiosis in plants, some abnormal meiosis phenomena will occur, such as meiosis occurring at the microspore tetrad stage where the emergence of the binary or ternary gametes provides the most direct evidence of 2n gamete formation [36]. Two types of 2n gametes, FDR and SDR, occur in plants. Theoretically, FDR-type and SDR-type gametes individually carry 80 and 40% of the heterozygosity from the parents. FDR-type gametes have one chromosome for every pair of homologous chromosomes, while SDR-type have two sibling chromatids for one chromosome from the homologous chromosomes, such as in lily [37]. In the FDR type, homologous chromosomes either do not show pairing and separation, or the frequency of pairing is very low and the chromosomes occur in the univalent form during meiosis [38]. The two sibling chromatids of homologous chromosomes move to opposite poles during the second division. FDR-type gametes hold all the genes of the parents except for the cross-exchanged fragments. In SDR-type gametes, homologous chromosome pairing and separation is normal in the first meiotic division, although the centromeres divide abnormally during the second meiotic division, with the result that the chromatids do not move towards the poles. In that case, only half of the parental chromosomes occur in the SDR gametes and these chromosomes have two copies, such as in *Begonia* [39]. According to their genetic influence, the occurrence of FDR-type 2n gametes is usually the result of mutation and abnormal spindles, while SDR-type 2n gametes are the result of abnormal cytokinesis. High heterozygosity of the FDR-type gametes determines their ability to transfer parent heterozygosity and epistatic efficiently, and therefore have a higher utilization value in breeding. The production of 2n pollen based on a series of meiosis observations that focused on FDR-types was previously discussed, including the cellular processes of the dyad products of the parallel spindles and the triad products of the vertical spindles [29, 31, 34]. The hybridization offspring of two lily cultivars (*L. auratum* × *L. henryi*) produced a number of 2n gametes and were crossed with another lily hybrid (the Oriental hybrid) to produce triploid offspring [40]. The self-pollinated offspring of the diploid *Citrus tamurana* ‘Nishiuchi Konatsu’ are tetraploids, whereas other combinations of the offspring are diploids [41]. It has been shown that FDR-types form the majority, while IMR (indeterminate meiotic restitution)-types are the minority based on the number of 2n gametes produced by genomic offspring through in situ hybridization (GISH) in lily [37]. Plant autopolyploids or allopolyploids can be obtained by the induction of 2n gametes, and making better use of their genetic diversity and heterosis in *Arabidopsis* [42].

During microsporogenesis, abnormal meiotic behavior is related to the formation of 2n pollen, such as an in asynapsis [43], the distribution of microtubular cytoskeletons and organelle nucleoids in hybrid *Populus* [44], as well as deviations in the spatial configuration of both MT (microtubule)s and MF (microfilament)s of *Solanum* [45]. Furthermore, the production of 2n pollen in plants is mainly controlled by genes. An allelic meiosis-related gene *Jason* was previously cloned and is responsible for ploidy regulation during the process of 2n gametogenesis in *A. thaliana* [7]. The *OSDl* gene is responsible for initiating the second division of meiosis, forming SDR-type 2n male and female gametes [46]. The *SWITCH1/DYAD* gene has the potential to induce 2n gamete production in *A. thaliana* [47]. In a weak *SWI* gene mutant *DYAD* of *A. thaliana*, sibling chromatid pairing was disturbed and synapsis failed, producing a non-meiosis female gamete. Further experiments have shown that these female gametes without meiosis can cross with normal 1n pollen, creating triploid offspring [48]. The abnormal callose accumulation led to cell plate formation in defective cells and produced 2n gametes in telophase II of meiosis in the tomato mutant *pmcd1* [49]. *DcPS1* genes probably play a role in 2n pollen formation in carnations (*Dianthus caryophyllus*) as a response to temperature stress, which is attributable to abnormal spindle orientation at male meiosis II [50].

Several tetraploid species of *Brachiaria* can naturally produce FDR 2n pollen [36], while asynchronous meiosis was observed in both sexually reproducing diploid species and apomixes tetraploid species [51], as observed in ‘Old Blush’. As a successive type of meiosis, potatoes produce normal dyads, and chromosome bridges and lagging chromosomes were found in anaphase I and II [52]. A parallel spindle resulted in abnormal meiosis in rose [29, 34], whereas other studies suggest that the parallel spindle is a common process in the formation of normal tetrads, and a fused spindle indicates abnormal meiosis [53]. In our study, the fusion spindle and the triple spindle developed from the vertical spindle, and thus it was proposed that the 2n pollen was mainly generated from two abnormal division methods in rose.

### Competitive advantage of 2n pollen in *R. hybrida*

Many hybrid combinations between tetraploid and diploid roses produce a relatively high number (> 25%) of tetraploids, and it is believed that 2n pollen is likely to have a selective advantage in the pollination process [33]. Our experiment revealed that the natural rate of 2n gamete production is about 1.39%. At such a low rate of production of 2n gametes, there will still be some tetraploid progeny in each hybrid combination, suggesting that 2n pollen has considerable competitive advantage against normal 1n pollen production. It remains uncertain whether the performance of 2n pollen indicates greater viability than that of 1n pollen, or whether 2n pollen tubes extend faster than 1n pollen tubes on stigmas. Based on this, we recommend the further exploration of these questions in future studies.

Furthermore, it should be noted that a large number of cabbage cultivars are generally diploid. However, tetraploid production was induced by colchicine or by the natural sexual mating of 2n pollen. Tetraploids have the advantage of high quality, good flavor, and high yield compared with ordinary diploids in *Brassica pekinensis,* which also suggests that 2n gametes have certain competitive advantages over 1n gametes [54]. In most angiosperms, 2n gametes are disadvantaged against 1n gametes in double fertilization, as is the case, for example, in *Populus tomentosa*. However, while 2n pollen has a potential germination ability, their germination process is relatively slow compared with 1n pollen and is disadvantaged against 1n gametes when participating in fertilization, which is the primary explanation for the low breeding rate of triploids [55].

1n and 2n pollen on the stigmas of ‘Dien’ and ‘Phuc Trach’ Pummelo (*Citrus grandis*) all germinate normally after pollination; however, compared with 1n pollen, 2n pollen germinates later and the pollen tubes extend more slowly and are thus disadvantaged in their ability to compete in fertilization [56]. In some plants, 2n gametes are relatively strong. For example, the *Solanum phureja* gametes without meiosis have greater viability than those exhibiting normal meiosis [57]. The pollen tubes of 2n pollen grow faster than those of 1n pollens on the stigmas of *Solanum tuberosum* [58].

### Relationship between ploidy and pollen size

In our study, the diameter of the natural 2n pollen was about 30% larger in size than the 1n pollen. It is well known that pollen size increases with increasing DNA [59]. The pollen diameters of various *Phalaenopsis* polyploids are positively correlated. A positive correlation (r = 0.935, *P* < 0.065) between ploidy and pollen diameters was found by Zhu et al. [60]. The average size of the pollen grains (diploid pollen) of tetraploid species is 1.3 times greater than that of diploid species (haploid pollen) [28, 30, 33], providing a direct method for estimating 2n pollen by their size. Pollen size can influence the relative siring success of different individuals competing on the same stigma during post-pollination processes in *Ipomoea purpurea* [61]. Larger pollen has greater competitive potential than normal sized pollen.

### The germination and growth of 2n pollen in ‘old blush’ and fructification characters in the pistil

While the germination strength was not obviously stronger, the proportion, length, and growth rate of 2n pollen tube gradually exceeded 1n pollen tube through competitive advantage in the stigma internal transmitting tissue, and the 2n pollen possessed strong competitive advantage during fertilization. Thus, the strong competitive advantage of the 2n pollen was not decided by the pollen grain itself, but has a close interaction with pistil selection. The critical period of the 2n pollen plays a role in its competitive advantage when traversing the internal transmitting tissue of the stigma. For most pathways, the anatomical structure of the tissues can restrict the growth of pollen tubes mechanically, and lipids, water, and glycosylated proteins in the pistil tissue are required as supplementation, including for the provision of signal guidance [62]. The binding of STIGMA-SPECIFIC PROTEIN1 (STIG1) with the pollen receptor kinase LePRK2 can promote pollen tube growth in vitro, and both the pollen tube elongation rate and seed production were reduced in vivo in a STIG1 mutant in tomato (*Solanum lycopersicum*) [63]. Glutamate receptor-like channels (GLRs) facilitate Ca^2+^ influx across the plasma membrane and modulate the apical calcium concentration gradient, which is a key factor influencing pollen tube growth and morphogenesis [64]. Egg-apparatus secreting the 94-aa peptide ZmEA1 in maize were required for guiding the pollen tube growth before double fertilization. When it was down-regulated, the pollen tube would lose guidance and resulted in abortion [65]. Using two-photon excitation (TPE) stimulation microscope, it was further confirmed that MYB98 and ZmEA1 respond to Ca^2+^ and NO signals and play an important role in the guidance of the pollen tube in *Arabidopsis* [66].

Callose plugs in angiosperm pollen tubes play a role in locating the cytoplasm and reproductive units at the apical pollen tube and separating the apical region, so that the reproductive unit can be kept far away from the active region in the apex of the pollen tube [67, 68]. In the pollen tube of some genotypes of *A. thaliana*, the callose plug deposited thicker along with the pollen tube growth period [69]. We can thus speculate that a thinner callose plug may result in higher pollen tube activity and weaker hindrance to the movement of the reproductive unit, while a callose plug with a lower density would result in a faster pollen tube growth rate. It was also observed that the thickness and density of the callose plug in 2n pollen tubes generally differed significantly from the 1n pollen tube, and were associated with pollen competitive advantage. The cell wall of pollen tube is mainly composed of polysaccharides with pectin, cellulose, and callose, which affect cell stiffness and the viscoelasticity of the pollen grains, and provides resistance to the circumferential tension stress of the pollen tube [70]. In addition, the callose periodically deposits and forms a callose plug in order to maintain the apical expansion area of the pollen tube and separate the active region from the degraded areas. A slow-growing pollen tube will not continue to present a stable callose on the cell wall and there will be no deposition of the callose plug [71]. Callose consists of β-l,3-glucan, and the latter is synthesized via callose synthase (CalS), which is in the plasmalemma. Complexes are deposited in the paramural space between the pre-existing cellulosic cell wall and the plasma membrane [72]. This model was confirmed using dSTORM-dyed by S4B and ABF dye solution [73, 74]. The synthesis of pollen callose is closely related to the regulation of pollen tube growth. Pollen tube growth depends on the polarized plasma membrane transporters, calcium concentration gradient oscillations, and cooperation with other conductive mechanisms, which constitutes the central regulatory mechanism of pollen tube growth [75] and partakes in the downstream regulation of pollen tube growth, such as in NO signaling or LURE [62, 76, 77]. Treatment with the NO donor S-nitroso-N-acetylpenicillamine (SNAP) promoted NO signal transduction, extracellular Ca^2+^ influx, and increased the content of actin. The parallel elongation microfilament bundle was extended to the apex of the pollen tube, ultimately resulting in the significant increase in the configuration and distribution of callose synthase in the apical pollen tube and pollen tube growth, indicating that callose synthase is dependent on microfilament transport; a process regulated by calcium signaling [78]. The distribution of callose affects the normal growth of the pollen tube, and is closely related to the regulation of pollen tube growth. The morphology of the callose plug is the embodiment of pollen tube growth and is an important factor that affects the competitiveness of pollen.

## Conclusions

We conclude that 2n pollen is involved in hybridization where it possesses a competitive advantage. The results of the cytological observations indicated that 2n pollen developed from the defeated cytoplasmic division or nuclear division in the meiosis metaphase II of PMC (pollen mother cell) in ‘Old Blush’. The natural generation rate of the 2n pollen in ‘Old Blush’ (2x) was about 1.39%, whereas the tetraploids in the F1 offspring possessed a high rate, i.e., 44.00%. The temporal and spatial characteristics of ‘Old Blush’ pollen germination on the stigma and growth in pistil of ‘Orange Fire’ and ‘DEE’ were observed, and the results suggested that the germination rate of 2n pollen on the stigma was not superior to that of 1n pollen, but that the proportion of 2n pollen increased to 30.90 and 37.20%, respectively, while it traversed the stigma and entered into style. The callose plug in the 2n pollen tube was significantly thinner than that of 1n pollen tube, which may have strongly influenced the competitive process in *R. hybrida*. Each trait involved in our experiment is very important for F1 morphological phenotypes.

## Methods

### Hybrid experiments

The experiments on the four hybrid combinations, shown in Table 1, were carried out from May 2013 to July 2017. Two days prior to hybridization, the anthers from unopened flowers of ‘Old Blush’, i.e., the male parents, were collected in Petri dishes and then stored in a dry and ventilated area where the anthers cracked naturally and shed pollen. We peeled off the petals and all stamens, from the outside to the inside, from the unopened flowers of ‘Orange Fire’, i.e., the female parents in our hybridization experiment. The male pollen was placed onto the flowering female stigma, disposing of the entire set of stamens with a brush. We bagged and labeled the parchments after pollination. Twenty days later, the paper bags were removed from the inflorescences. Fruits and seeds were harvested on October 18, 2013 and placed in wet sand storage at 1–4 °C, after which they were removed from the sand and sown in a greenhouse at 25 °C. The germination rate was determined statistically. F1 seedlings were planted in an open field on June 1, 2014. The morphology of the two-year-old F1 plants was investigated in the period from May to October, 2015. The plant materials in the experiment were collected from and approved by the Beijing Institute of Landscape Architecture.

### Identification of ploidy by flow cytometry and compression method for the chromosomes of the stem tips

About 1.0 g of fresh leaves of each plant was used. The samples were washed with distilled water, dried with filter paper, and placed on a chilled Petri dish onto which pre-cooled LB01 lysis buffer (approximately 1–2 mL) was added, and the leaves were rapidly chopped once with a sharp blade. The samples were immersed in the lysis buffer during the entire process in order to efficiently dissociate the nuclei. The lysis buffer was absorbed by the samples from the dish and filtered into a 1.5 mL Eppendorf tube through a 400 μm mesh filter membrane and then placed in a refrigerator and incubated for 5 min at 4 °C. The samples were then centrifuged at 1500 r/min for 5 min at 4 °C. Then, 100 μL of chilled lysis buffer and 150 μL of chilled PI (Propidium Iodide) dye were added to each sample after discarding the supernatant. The samples were then stored in the dark in a refrigerator and stained for 10 min at 4 °C. The samples were the transferred to the sample tubes where, using flow cytometry (FCM) (BD Company Accuri C6 flow cytometry), we detected and collected 5000–10,000 particles. The known ploidy of ‘Old Blush’ was used as the external standard for ploidy, and the ploidy samples were determined by comparing the intensity of their fluorescent signals.

The tips of vigorous growth shoots were immersed in a saturated santochlor aqueous solution for 2 h, washed with water, and then fixed in a Kano fixative (ethanol: acetic acid = 3: 1) for a period of 2 h to 24 h, after which they were placed in a lysis buffer (hydrochloric acid: 95% ethanol = 1: 1) for 20–30 min at 20 °C. They were then washed for 15 min three times, compressed, and observed after dyeing with improved basic magenta.

### The natural rate of generation and viability of 2n pollen in ‘old blush’

A mixture of natural 1n pollen and 2n pollen in ‘Old Blush’ was collected and stained with 0.1% aceto-carmine, and the pollen diameter was measured with a Leica DM 2500 Fluorescence microscope using Digimizer image analysis software V 4.2.6. The experiment was repeated three times. We counted the number of pollens under a microscope and established the natural rate of generation of 2n pollen through 15 observations.

The 2n pollen of ‘Old Blush’ was identified using the method of Crespel et al. [29]. Briefly, the pollen diameters were measured after Alexander staining under the microscope, and more than 500 pollen grains were measured for each sample. The number of pollens of different sizes was counted using a gradient of 2 μm, so that the average diameter of the gradient with the largest number of pollens was considered as the average diameter of the 1n pollen population, and the diameter of the 2n pollen was more than 1.3 times that of the average diameter of the 1n pollen. The same method was used to calculate the pollen tube width and to evaluate whether there was a positive correlation between pollen tube width and pollen diameter at germination at 6 h in vitro*.* If a positive correlation was found, the criterion for determining the 2n pollen tube in the style could be used in our experiment.

### Cytological observations of 2n pollen generated in the ‘old blush’ diploid

Floral buds of the ‘Old Blush’ diploid were collected at different developmental stages, ranging from the pollen mother cells to mature pollen, washed, and fixed in a Kano fixative (ethanol-acetic solution) for 2–24 h. These were then dissociated in a solution of hydrochloric acid (95% ethanol = 1, 1) for 20–30 min at room temperature and again dissociated and washed three times for 15 min each time. The pollen were stripped from the flower buds and stained with 0.10% aceto-carmine, compressed, and observed with a Leica DM 2500 and a Leica DFC 500 microscope. In order to study the ultrastructure, the pollen of ‘Old Blush’ was fastened to the microscope stage using special adhesive tape and observed with a scanning electron microscope (HITACHI TM3000).

### Molecular identification of genuine hybrids of F1 offspring using SSR and SRAP molecular markers

Genomic DNA was extracted using a DP320 plant genome extraction kit (Tiangen Bioch. Tech. Com., Beijing, China). The samples were separated by electrophoresis on 0.8% (*w*/*v*) agarose gels, with the quality and concentration of DNA was assessed using a UV spectrophotometer. These were then diluted to 25 ng/μL and stored in a refrigerator at − 20 °C during our experiments.

Using the genomic DNA as a template in the SSR experiment and the female parents of ‘Orange Fire’ and the male parents of ‘Old Blush’, 21 pairs of primers (Additional file 2: Table S5) were used for amplification, the polymorphic primers in parents were screened for SSR amplification of the F1 hybrid progeny. The primer combinations were designed, screened, and synthesized by the Beijing Ruibo Xingke (www.ruibiotech.com) Biological Technology Ltd., Co..

In the SRAP experiment, a total of 14 pair of primers of upstream and downstream primer combinations were designed and screened and then synthesized by the Shanghai Biological Engineering Technology Services Ltd., Co. One pair of primer combinations with clear and stable amplified bands was screened out and used in the formal amplification.

Each 25 μL PCR mixture consisted of 12.5 μL 2 × Taq PCR MasterMix, 1 μL of a primer solution, 1 μL template solution, and 10.5 μL of ddH_2_O. The following PCR conditions were used: initial denaturation at 94 °C for 5 min, followed by five cycles of denaturation at 94 °C for 1 min, annealing at 35 °C for 1 min, extension at 72 °C for 1 min, and then 30 cycles of denaturation at 94 °C for 1 min, annealing at 50 °C for 1 min, extension at 72 °C for 1 min, and a final extension at 72 °C for 10 min. The samples were separated by electrophoresis on 2% (w/v) agarose gels. Each gel electrophoresis was run at a constant voltage of 150 V at 20 °C for 30 min and then observed and photographed using a gel imaging system.

### The temporal and spatial characteristics of ‘old blush’ pollen germination on the stigma, and growth in the pistil of ‘Orange fire’ and ‘DEE’

Both the ‘Orange Fire’ and ‘DEE’ female parent flowers were collected at 0 h, 4 h, 6 h, 8 h, 12 h, 16 h, 20 h, 24 h, 48 h, and 72 h after pollination of ‘Old Blush’ pollen respectively, and the intact carpels were stripped and stored in FAA (formalin-acetic acid-alcohol) solution overnight at 4 °C. The temporal and spatial characteristics of ‘Old Blush’ 1n and 2n pollen germination on the stigma, pollen tube movement and elongation, and ovary entry of ‘Orange Fire’ and ‘DEE’ were observed under the UV light of the fluorescent microscope after softening and staining with aniline blue. In combination with the seed setting rate statistical results, the 2n pollen competition advantage was confirmed. Three buds were used in each treatment, and 10 pollinated pistils were observed in each sample.

### Principal component analysis of the genetic traits

Spss statistics v17.0 software was used for statistics of Principal component analysis PCA), which was used to analyze the nine characteristic traits, i.e., ploidy, plant type, color, petals, flower diameter, the top leaflet length, the top leaflet width, leaflet shape, and aculeus of the ‘Orange Fire’ × ‘Old Blush’ F1 hybrids. We further investigated the correlation between the four phenotypic quantitative traits, i.e., flower diameter, length of the top leaflet, length of the pollen polar axis, and length of the guard cells.

## Additional files

Additional file 1: **Figure S1.** Somatic cell chromosomes of stem tips and chromosome ploidy pictures of F1 hybrids of ‘Orange Fire’ × ‘Old Blush’. A1. flower of No.4 F1 (2n = 3x = 21) from ‘Orange Fire’ × ‘Old Blush’. A2. somatic cell chromosomes of No.4 F1. A3. chromosome ploidy picture of No.4 F1 detected by flow cytometry with a fluorescence intensity of 361,282. B1. flower of No.5 F1 (2n = 4x = 28) from ‘Orange Fire’ × ‘Old Blush’. B2. somatic cell chromosomes of No.5. B3. chromosome ploidy picture of No.5 F1 detected by flow cytometry with a fluorescence intensity of 489,697. C1.flower of No.7 F1 (2n = 4x = 28) from ‘Orange Fire’ × ‘Old Blush’. C2. somatic cell chromosomes of No.7 F1. C3. chromosome ploidy picture of No.7 F1 detected by flow cytometry with a fluorescence intensity of 452,313. D1.flower of No.11 F1 (2n = 4x = 28) from ‘Orange Fire’ × ‘Old Blush’. D2. somatic cell chromosomes of No.11 F1. D3. chromosome ploidy picture of No.11 F1 detected by flow cytometry with a fluorescence intensity of 533,757. E1. flower of No.12 F1 (2n = 4x = 28) from ‘Orange Fire’ × ‘Old Blush’. E2. somatic cell chromosomes of No.12 F1. E3. chromosome ploidy picture of No.12 F1 detected by flow cytometry with a fluorescence intensity of 479,809. F1. flower of No.14 F1 (2n = 4x = 28) from ‘Orange Fire’ × ‘Old Blush’. F2. somatic cell chromosomes of No.14 F1. F3. chromosome ploidy picture of No.14 F1 detected by flow cytometry with a fluorescence intensity of 511,126. G1. flower of No.16 F1 (2n = 3x = 21) from ‘Orange Fire’ × ‘Old Blush’. G2. somatic cell chromosomes of No.16 F1. G3. chromosome ploidy picture of No.16 F1 detected by flow cytometry with a fluorescence intensity of 369,934. H1. flower of No.17 F1 (2n = 3x = 21) from ‘Orange Fire’ × ‘Old Blush’. H2. somatic cell chromosomes of No.17 F1. H3. chromosome ploidy picture of No.17 F1 detected by flow cytometry with a fluorescence intensity of 420,158. I1. flower of No.18 F1 (2n = 3x = 21) from ‘Orange Fire’ × ‘Old Blush’. I2. somatic cell chromosomes of No.18 F1. I3. chromosome ploidy picture of No.18 F1 detected by flow cytometry with a fluorescence intensity of 424,627. J1. flower of No.19 F1 (2n = 3x = 22) from ‘Orange Fire’ × ‘Old Blush’. J2. somatic cell chromosomes of No.19 F1. J3. chromosome ploidy picture of No.19 F1 detected by flow cytometry with a fluorescence intensity of 404,424. K1. flower of No.24 F1 (2n = 4x = 28) from ‘Orange Fire’ × ‘Old Blush’. K2. somatic cell chromosomes of No.24 F1. K3. chromosome ploidy picture of No.24 F1 detected by flow cytometry with a fluorescence intensity of 494,438. L1. flower of No.25 F1 (2n = 4x = 28) from ‘Orange Fire’ × ‘Old Blush’. L2. somatic cell chromosomes of No.25 F1. L3. chromosome ploidy picture of No.25 F1 detected by flow cytometry with a fluorescence intensity of 459,819. **Figure S2.** Ploidy diagrams of parents and F1 hybrids of ‘Orange Fire’ × ‘Old Blush’ detected by flow cytometry. **Figure S3.** Ploidy diagrams of parents and F1 hybrids of ‘Chun Chao’ × ‘Slater’s Crimson China’ detected by flow cytometry. **Figure S4.** Ploidy diagrams of parents and F1 hybrids of ‘DEE’ × ‘Slater’s Crimson China’ detected by flow cytometry. **Figure S5.** Ploidy diagrams of parents and F1 hybrids of ‘DEE’ × ‘Old Blush’ detected by flow cytometry. (DOC 2564 kb)

Additional file 2 **Table S1.** The DNA amounts of parents and F1 hybrids of ‘Orange Fire’ × ‘Old Blush’ detected by flow cytometry. **Table S2.** The DNA amounts of parents and F1 hybrids of ‘Chun Chao’ × ‘Slater’s Crimson China’ detected by flow cytometry. **Table S3.** The DNA amounts of parents and F1 hybrids of ‘DEE’ × ‘Slater’s Crimson China’ detected by flow cytometry. **Table S4.** The DNA amounts of parents and F1 hybrids of ‘DEE’ × ‘Old Blush’ detected by flow cytometry. **Table S5.** The 21 pairs of rose SSR primers used in paternity test. (DOCX 29 kb)

## Abbreviations

AFLP: Amplified fragment length polymorphism
AFLP: Amplified fragment length polymorphisms
CalS: Callose synthase
F1: The first filial generation
FAA: Formalin-acetic acid-alcohol
FCM: Flow cytometry
FCM: Flow cytometry
FDR: First division restitution
GISH: Genomic in situ hybridization
GLRs: Glutamate receptor-like channels
IMR: Indeterminate division restitution
MF: Microfilament
MT: Microtubule
PCR: Polymerase chain reaction
PI: Propidium Iodide
PMC: Microspore mother cells
SDR: Second division restitution
SDR: Second division restitution
SNAP: S-nitroso-N-acetylpenicillamine
SRAP: Sequence-related am-plified polymorphism
SSR: Simple sequence repeat
STIG1: STIGMA-SPECIFIC PROTEIN1
UV: Ultraviolet
