# Supplementary material for: The strong competitive role of 2n pollen in several polyploidy hybridizations in Rosa hybrida
Source: BMC Plant Biol. 2019 Apr 4;19:127. doi: 10.1186/s12870-019-1696-z (PMC6449914; doi:10.1186/s12870-019-1696-z)
Supplement: Supplementary file 1 — Figure S1. Somatic cell chromosomes of stem tips and chromosome ploidy pictures of F1 hybrids of ‘Orange Fire’ × ‘Old Blush’. A1. flower of No.4 F1 (2n = 3x = 21) from ‘Orange Fire’ × ‘Old Blush’. A2. somatic cell chromosomes of No.4 F1. A3. chromosome ploidy picture of No.4 F1 detected by flow cytometry with a fluorescence intensity of 361,282. B1. flower of No.5 F1 (2n = 4x = 28) from ‘Orange Fire’ × ‘Old Blush’. B2. somatic cell chromosomes of No.5. B3. chromosome ploidy picture of No.5 F1 detected by flow cytometry with a fluorescence intensity of 489,697. C1.flower of No.7 F1 (2n = 4x = 28) from ‘Orange Fire’ × ‘Old Blush’. C2. somatic cell chromosomes of No.7 F1. C3. chromosome ploidy picture of No.7 F1 detected by flow cytometry with a fluorescence intensity of 452,313. D1.flower of No.11 F1 (2n = 4x = 28) from ‘Orange Fire’ × ‘Old Blush’. D2. somatic cell chromosomes of No.11 F1. D3. chromosome ploidy picture of No.11 F1 detected by flow cytometry with a fluorescence intensity of 533,757. E1. flower of No.12 F1 (2n = 4x = 28) from ‘Orange Fire’ × ‘Old Blush’. E2. somatic cell chromosomes of No.12 F1. E3. chromosome ploidy picture of No.12 F1 detected by flow cytometry with a fluorescence intensity of 479,809. F1. flower of No.14 F1 (2n = 4x = 28) from ‘Orange Fire’ × ‘Old Blush’. F2. somatic cell chromosomes of No.14 F1. F3. chromosome ploidy picture of No.14 F1 detected by flow cytometry with a fluorescence intensity of 511,126. G1. flower of No.16 F1 (2n = 3x = 21) from ‘Orange Fire’ × ‘Old Blush’. G2. somatic cell chromosomes of No.16 F1. G3. chromosome ploidy picture of No.16 F1 detected by flow cytometry with a fluorescence intensity of 369,934. H1. flower of No.17 F1 (2n = 3x = 21) from ‘Orange Fire’ × ‘Old Blush’. H2. somatic cell chromosomes of No.17 F1. H3. chromosome ploidy picture of No.17 F1 detected by flow cytometry with a fluorescence intensity of 420,158. I1. flower of No.18 F1 (2n = 3x = 21) from ‘Orange Fire’ × ‘Old Blush’. I2. somatic cell [file 12870_2019_1696_MOESM1_ESM.doc]

**Supplementary data**

Figure legends

**Figure S1. Somatic cell chromosomes of stem tips and chromosome ploidy pictures of F1 hybrids of ‘Orange Fire’ × ‘Old Blush’**

A1. flower of No.4 F1 (2n=3x=21) from ‘Orange Fire’× ‘Old Blush’. A2. somatic cell chromosomes of No.4 F1. A3. chromosome ploidy picture of No.4 F1 detected by flow cytometry with a fluorescence intensity of 361282. B1. flower of No.5 F1 (2n=4x=28) from ‘Orange Fire’× ‘Old Blush’. B2. somatic cell chromosomes of No.5. B3. chromosome ploidy picture of No.5 F1 detected by flow cytometry with a fluorescence intensity of 489697. C1.flower of No.7 F1 (2n=4x=28) from ‘Orange Fire’× ‘Old Blush’. C2. somatic cell chromosomes of No.7 F1. C3. chromosome ploidy picture of No.7 F1 detected by flow cytometry with a fluorescence intensity of 452313. D1.flower of No.11 F1 (2n=4x=28) from ‘Orange Fire’× ‘Old Blush’. D2. somatic cell chromosomes of No.11 F1. D3. chromosome ploidy picture of No.11 F1 detected by flow cytometry with a fluorescence intensity of 533757. E1. flower of No.12 F1 (2n=4x=28) from ‘Orange Fire’× ‘Old Blush’. E2. somatic cell chromosomes of No.12 F1. E3. chromosome ploidy picture of No.12 F1 detected by flow cytometry with a fluorescence intensity of 479809. F1. flower of No.14 F1 (2n=4x=28) from ‘Orange Fire’× ‘Old Blush’. F2. somatic cell chromosomes of No.14 F1. F3. chromosome ploidy picture of No.14 F1 detected by flow cytometry with a fluorescence intensity of 511126. G1. flower of No.16 F1 (2n=3x=21) from ‘Orange Fire’× ‘Old Blush’. G2. somatic cell chromosomes of No.16 F1. G3. chromosome ploidy picture of No.16 F1 detected by flow cytometry with a fluorescence intensity of 369934. H1. flower of No.17 F1 (2n=3x=21) from ‘Orange Fire’× ‘Old Blush’. H2. somatic cell chromosomes of No.17 F1. H3. chromosome ploidy picture of No.17 F1 detected by flow cytometry with a fluorescence intensity of 420158. I1. flower of No.18 F1 (2n=3x=21) from ‘Orange Fire’× ‘Old Blush’. I2. somatic cell chromosomes of No.18 F1. I3. chromosome ploidy picture of No.18 F1 detected by flow cytometry with a fluorescence intensity of 424627. J1. flower of No.19 F1 (2n=3x=22) from ‘Orange Fire’× ‘Old Blush’. J2. somatic cell chromosomes of No.19 F1. J3. chromosome ploidy picture of No.19 F1 detected by flow cytometry with a fluorescence intensity of 404424. K1. flower of No.24 F1 (2n=4x=28) from ‘Orange Fire’× ‘Old Blush’. K2. somatic cell chromosomes of No.24 F1. K3. chromosome ploidy picture of No.24 F1 detected by flow cytometry with a fluorescence intensity of 494438. L1. flower of No.25 F1 (2n=4x=28) from ‘Orange Fire’× ‘Old Blush’. L2. somatic cell chromosomes of No.25 F1. L3. chromosome ploidy picture of No.25 F1 detected by flow cytometry with a fluorescence intensity of 459819.

**Figure S2. Ploidy diagrams of parents and F1 hybrids of ‘Orange Fire’ × ‘Old Blush’ detected by flow cytometry**

**Figure S3. Ploidy diagrams of parents and F1 hybrids of ‘Chun Chao’ × ‘Slater`s Crimson China’ detected by flow cytometry**

**Figure S4. Ploidy diagrams of parents and F1 hybrids of ‘DEE’ × ‘Slater`s Crimson China’ detected by flow cytometry**

**Figure S5. Ploidy diagrams of parents and F1 hybrids of ‘DEE’ × ‘Old Blush’ detected by flow cytometry**


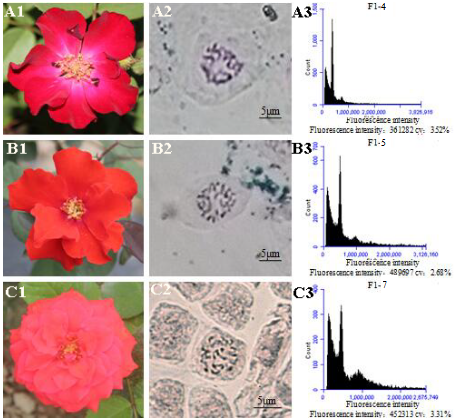

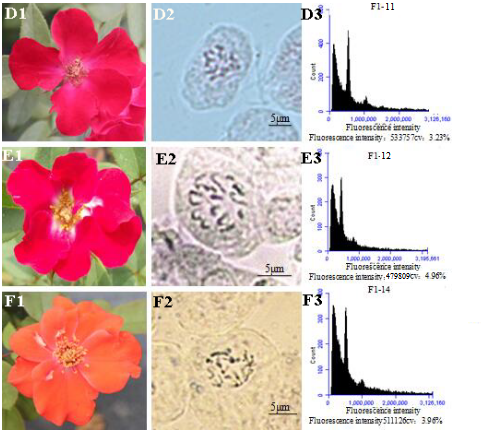


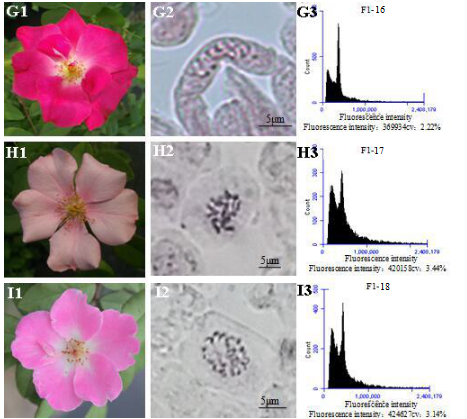


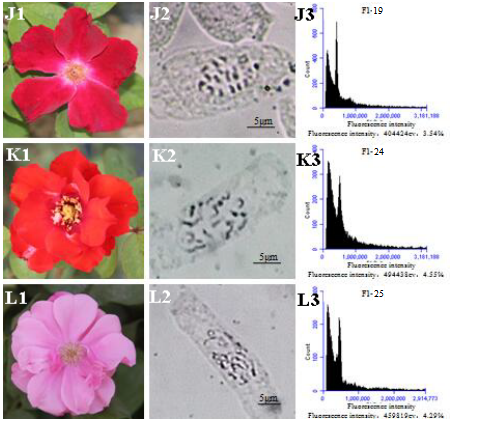


**Figure S1. Somatic cell chromosomes of stem tips and chromosome ploidy pictures of F1 hybrids of ‘Orange Fire’ × ‘Old Blush’**


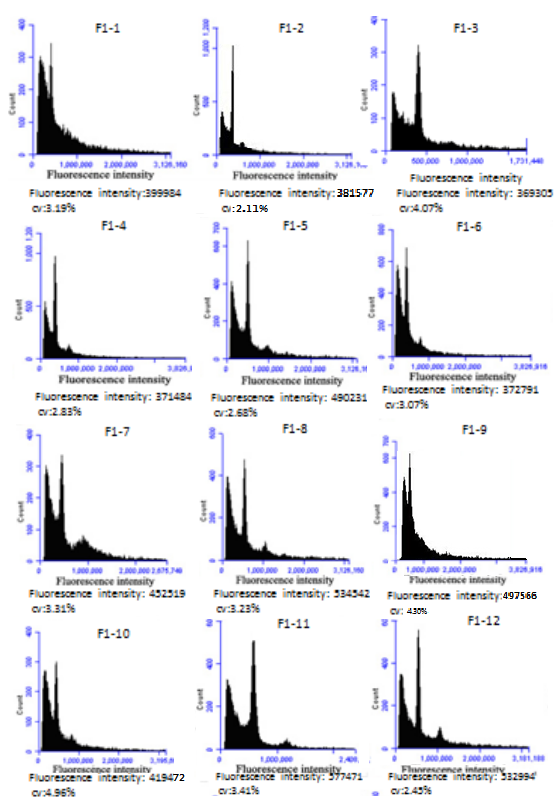


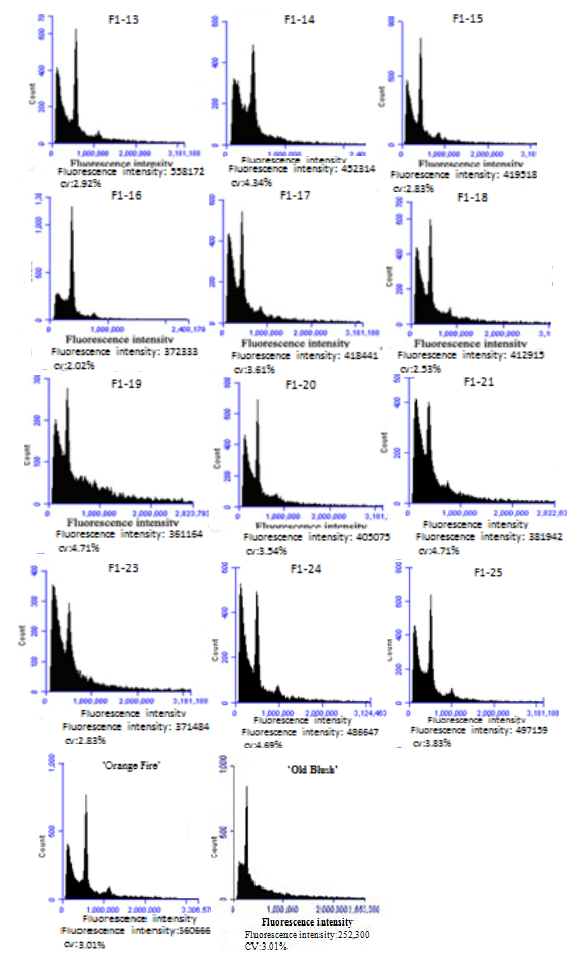


**Figure S2. Ploidy diagrams of parents and F1 hybrids of ‘Orange Fire’ × ‘Old Blush’ detected by flow cytometry**

**
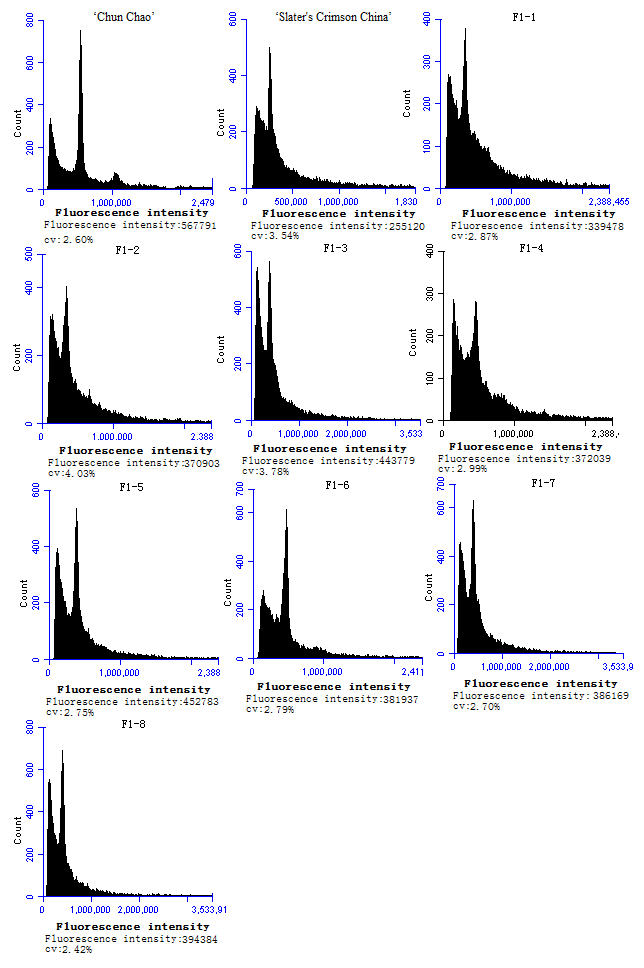
**

**Figure S3. Ploidy diagrams of parents and F1 hybrids of ‘Chun Chao’ × ‘Slater`s Crimson China’ detected by flow cytometry**

**
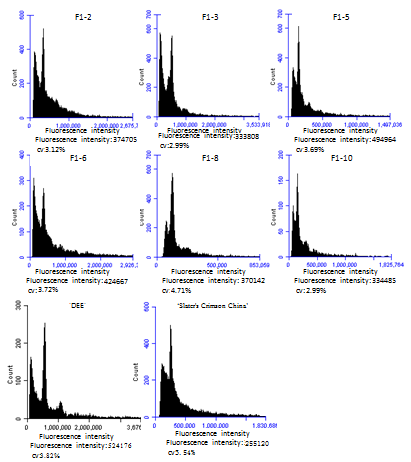
**

**Figure S4. Ploidy diagrams of parents and F1 hybrids of ‘DEE’ × ‘Slater`s Crimson China’ detected by flow cytometry**


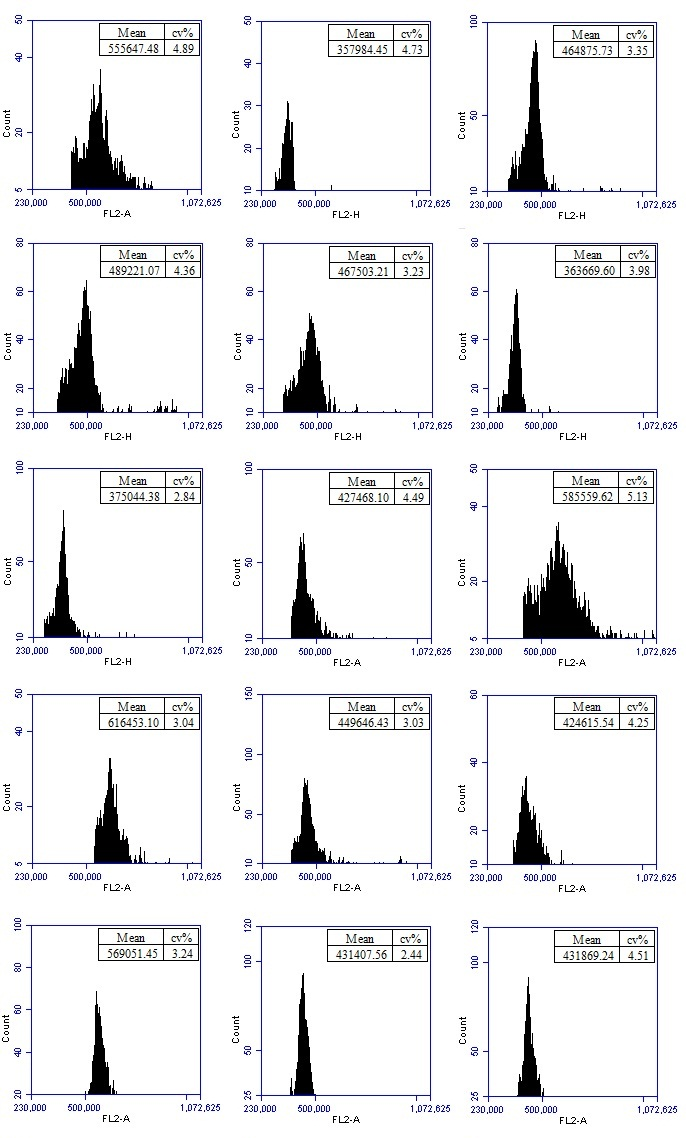


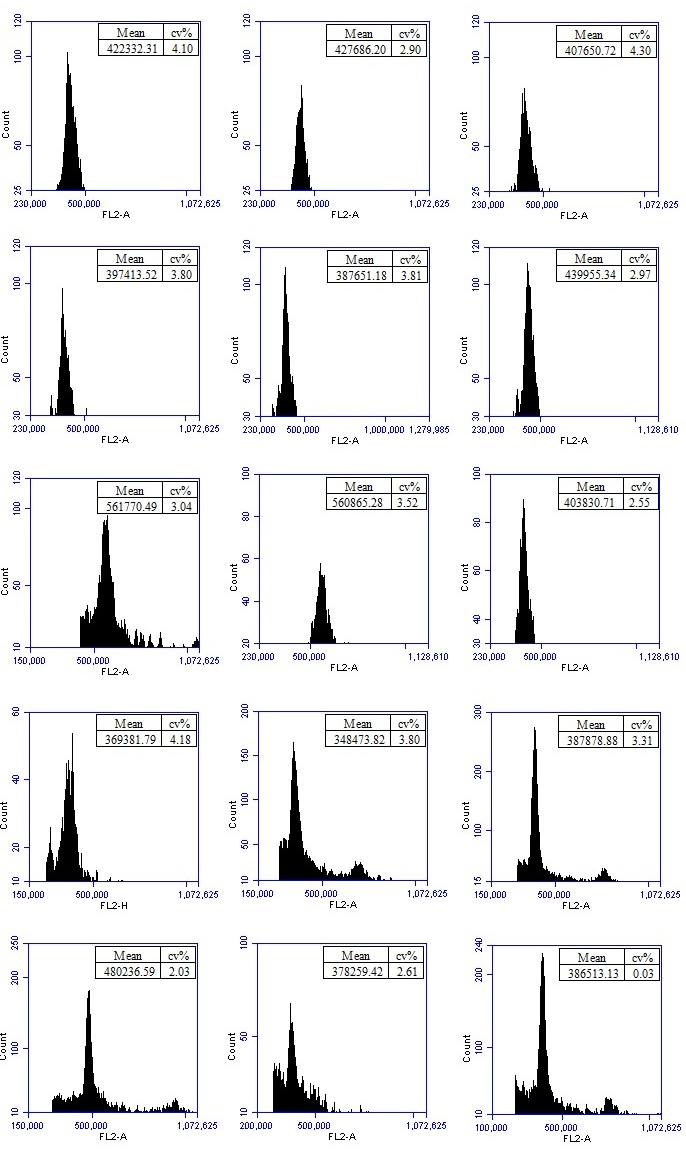


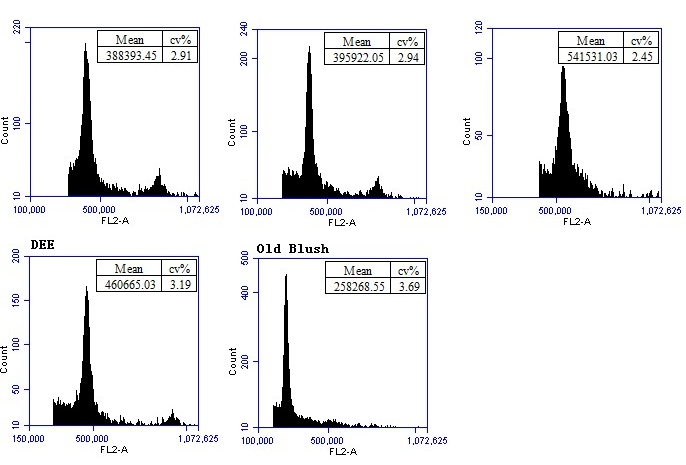


**Figure S5. Ploidy diagrams of parents and F1 hybrids of ‘DEE’ × ‘Old Blush’ detected by flow cytometry**
