# Supplementary material for: The strong competitive role of 2n pollen in several polyploidy hybridizations in Rosa hybrida
Source: BMC Plant Biol. 2019 Apr 4;19:127. doi: 10.1186/s12870-019-1696-z (PMC6449914; doi:10.1186/s12870-019-1696-z)
Supplement: Supplementary file 2 — Table S1. The DNA amounts of parents and F1 hybrids of ‘Orange Fire’ × ‘Old Blush’ detected by flow cytometry. Table S2. The DNA amounts of parents and F1 hybrids of ‘Chun Chao’ × ‘Slater’s Crimson China’ detected by flow cytometry. Table S3. The DNA amounts of parents and F1 hybrids of ‘DEE’ × ‘Slater’s Crimson China’ detected by flow cytometry. Table S4. The DNA amounts of parents and F1 hybrids of ‘DEE’ × ‘Old Blush’ detected by flow cytometry. Table S5. The 21 pairs of rose SSR primers used in paternity test. (DOCX 29 kb) [file 12870_2019_1696_MOESM2_ESM.docx]

**Table S1.** The DNA amounts of parents and F1 hybrids of ‘Orange Fire’ × ‘Old Blush’ detected by flow cytometry

| Number | Name of plant | Mean value of fluorescence light | CV/% | 2C DNA amounts | Ploidy | 1Cx-values/pg |
| --- | --- | --- | --- | --- | --- | --- |
| 1 | ‘Old Blush’ | 252300 | 3.01 | 1.42±0.02 | 2x=14 | 0.71±0.01 |
| 2 | ‘Orange Fire’ | 560660 | 3.01 | 2.82 | 4x=28 | 0.79 |
| 3 | F1-1 | 399984 | 3.19 | 2.25 | 3x=21 | 0.75 |
| 4 | F1-2 | 381577 | 2.11 | 2.15 | 3x=21 | 0.72 |
| 5 | F1-3 | 369303 | 4.07 | 2.08 | 3x=21 | 0.69 |
| 6 | F1-4 | 371484 | 2.83 | 2.09 | 3x=21 | 0.70 |
| 7 | F1-5 | 490231 | 2.68 | 2.76 | 4x=28 | 0.69 |
| 8 | F1-6 | 372791 | 3.07 | 2.10 | 3x=21 | 0.70 |
| 9 | F1-7 | 452519 | 3.31 | 2.55 | 4x=28 | 0.64 |
| 10 | F1-8 | 534542 | 3.23 | 3.01 | 4x=28 | 0.75 |
| 11 | F1-9 | 497566 | 4.3 | 2.80 | 4x=28 | 0.70 |
| 12 | F1-10 | 419472 | 4.96 | 2.36 | 3x=21 | 0.79 |
| 13 | F1-11 | 577471 | 3.41 | 3.25 | 4x=28 | 0.81 |
| 14 | F1-12 | 532994 | 2.45 | 3.00 | 4x=28 | 0.75 |
| 15 | F1-13 | 558172 | 2.92 | 3.14 | 4x=28 | 0.79 |
| 16 | F1-14 | 452314 | 4.34 | 2.55 | 4x=28 | 0.64 |
| 17 | F1-15 | 419518 | 2.83 | 2.36 | 3x=21 | 0.79 |
| 18 | F1-16 | 372333 | 2.02 | 2.10 | 3x=21 | 0.70 |
| 19 | F1-17 | 418441 | 3.61 | 2.36 | 3x=21 | 0.79 |
| 20 | F1-18 | 412915 | 2.53 | 2.32 | 3x=21 | 0.77 |
| 21 | F1-19 | 361164 | 4.71 | 2.03 | 3x=21 | 0.68 |
| 22 | F1-20 | 405075 | 3.54 | 2.28 | 3x=21 | 0.76 |
| 23 | F1-21 | 381942 | 4.71 | 2.15 | 3x=21 | 0.72 |
| 24 | F1-22 | 371484 | 2.83 | 2.09 | 3x=21 | 0.70 |
| 25 | F1-23 | 486647 | 4.69 | 2.74 | 4x=28 | 0.68 |
| 26 | F1-24 | 497159 | 3.83 | 2.80 | 4x=28 | 0.70 |
| 27 | F1-25 | 457936 | 4.29 | 2.58 | 4x=28 | 0.64 |

**Table S2.** The DNA amounts of parents and F1 hybrids of ‘Chun Chao’ × ‘Slater's Crimson China’ detected by flow cytometry

| Number | Name of plant | Mean value of fluorescence light | | CV/% | 2C DNA amounts | Ploidy | 1Cx-values/pg |
| --- | --- | --- | --- | --- | --- | --- | --- |
| 1 | ‘Chun Chao’ | 567791 | 2.6 | | 3.16 | 4x=28 | 0.79 |
| 2 | ‘Slater's Crimson China’ | 255120 | 3.54 | | 1.42±0.02 | 2x=14 | 0.71±0.01 |
| 3 | F1-1 | 339478 | 2.87 | | 1.889537 | 3x=21 | 0.63 |
| 4 | F1-2 | 370903 | 4.03 | | 2.064449 | 3x=21 | 0.69 |
| 5 | F1-3 | 443779 | 3.78 | | 2.470078 | 4x=28 | 0.62 |
| 6 | F1-4 | 372039 | 2.99 | | 2.070772 | 3x=21 | 0.69 |
| 7 | F1-5 | 452783 | 2.75 | | 2.520194 | 4x=28 | 0.63 |
| 8 | F1-6 | 381937 | 2.79 | | 2.125864 | 3x=21 | 0.71 |
| 9 | F1-7 | 386169 | 2.7 | | 2.14942 | 3x=21 | 0.72 |
| 10 | F1-8 | 394384 | 2.42 | | 2.195145 | 3x=21 | 0.73 |

**Table S3.** The DNA amounts of parents and F1 hybrids of ‘DEE’ × ‘Slater's Crimson China’ detected by flow cytometry

| Number | Name of plant | Mean value of fluorescence light | CV/% | 2C DNA amounts | Ploidy | 1Cx-values/pg |
| --- | --- | --- | --- | --- | --- | --- |
| 1 | ‘DEE’ | 524176 | 3.82 | 2.92 | 4X=28 | 0.73 |
| 2 | ‘Slater's Crimson China’ | 255120 | 3.54 | 1.42±0.02 | 2x=14 | 0.71±0.01 |
| 3 | F1-2 | 374705 | 3.12 | 2.09 | 3X=21 | 0.70 |
| 4 | F1-3 | 333808 | 2.99 | 1.86 | 3X=21 | 0.62 |
| 5 | F1-5 | 494964 | 3.69 | 2.75 | 4X=28 | 0.69 |
| 6 | F1-6 | 424667 | 3.72 | 2.36 | 3X=21 | 0.79 |
| 7 | F1-8 | 370142 | 4.71 | 2.06 | 3X=21 | 0.69 |
| 8 | F1-10 | 334485 | 2.99 | 1.86 | 3X=21 | 0.62 |

**Table S4.** The DNA amounts of parents and F1 hybrids of ‘DEE’ × ‘Old Blush’ detected by flow cytometry

| Number | Name of plant | Mean value of fluorescence light | | CV/% | 2C DNA amounts | Ploidy | 1Cx-values/pg |
| --- | --- | --- | --- | --- | --- | --- | --- |
| 1 | ‘DEE’ | | 524176 | 3.82 | 2.92 | 4x=28 | 0.73 |
| 2 | ‘Old Blush’ | | 258268 | 3.69 | 1.42±0.02 | 2x=14 | 0.71±0.01 |
| 3 | F1-1 | | 555647 | 4.89 | 3.06 | 4x=28 | 0.76 |
| 4 | F1-2 | | 357984 | 4.73 | 1.97 | 3x=21 | 0.66 |
| 5 | F1-3 | | 464875 | 3.35 | 2.56 | 4x=28 | 0.64 |
| 6 | F1-4 | | 489221 | 4.36 | 2.69 | 4x=28 | 0.67 |
| 7 | F1-5 | | 467503 | 3.23 | 2.57 | 4x=28 | 0.64 |
| 8 | F1-6 | | 363669 | 3.98 | 2.00 | 3x=21 | 0.67 |
| 9 | F1-7 | | 375044 | 2.84 | 2.06 | 3x=21 | 0.69 |
| 10 | F1-8 | | 427468 | 4.49 | 2.35 | 3x=21 | 0.78 |
| 11 | F1-9 | | 585559 | 5.13 | 3.22 | 4x=28 | 0.80 |
| 12 | F1-10 | | 616453 | 3.04 | 3.39 | 4x=28 | 0.85 |
| 13 | F1-11 | | 449646 | 3.03 | 2.47 | 3x=21 | 0.82 |
| 14 | F1-12 | | 424615 | 4.25 | 2.33 | 3x=21 | 0.78 |
| 15 | F1-13 | | 569051 | 3.24 | 3.13 | 4x=28 | 0.78 |
| 16 | F1-14 | | 431407 | 2.44 | 2.37 | 3x=21 | 0.79 |
| 17 | F1-15 | | 431869 | 4.51 | 2.37 | 3x=21 | 0.79 |
| 18 | F1-16 | | 422332 | 4.1 | 2.32 | 3x=21 | 0.77 |
| 19 | F1-17 | | 427686 | 2.9 | 2.35 | 3x=21 | 0.78 |
| 20 | F1-18 | | 407650 | 4.3 | 2.24 | 3x=21 | 0.75 |
| 21 | F1-19 | | 397413 | 3.8 | 2.19 | 3x=21 | 0.73 |
| 22 | F1-20 | | 387651 | 3.81 | 2.13 | 3x=21 | 0.71 |
| 23 | F1-21 | | 439955 | 2.97 | 2.42 | 3x=21 | 0.81 |
| 24 | F1-22 | | 561770 | 3.04 | 3.09 | 4x=28 | 0.77 |
| 25 | F1-23 | | 560865 | 3.52 | 3.08 | 4x=28 | 0.77 |
| 26 | F1-24 | | 403830 | 2.55 | 2.22 | 3x=21 | 0.74 |
| 27 | F1-25 | | 369381 | 4.18 | 2.03 | 3x=21 | 0.68 |
| 28 | F1-26 | | 348473 | 3.8 | 1.92 | 3x=21 | 0.64 |
| 29 | F1-27 | | 387878 | 3.31 | 2.13 | 3x=21 | 0.71 |
| 30 | F1-28 | | 480236 | 2.03 | 2.64 | 4x=28 | 0.66 |
| 31 | F1-29 | | 378259 | 2.61 | 2.08 | 3x=21 | 0.69 |
| 32 | F1-30 | | 386513 | 0.03 | 2.13 | 3x=21 | 0.71 |
| 33 | F1-31 | | 388393 | 2.91 | 2.14 | 3x=21 | 0.71 |
| 34 | F1-32 | | 395922 | 2.94 | 2.18 | 3x=21 | 0.73 |
| 35 | F1-33 | | 541531 | 2.45 | 2.98 | 4x=28 | 0.74 |

**Table S5.** The 21 pairs of rose SSR primers used in paternity test

| SSR Number | Positive(5’-3’) | Reverse(3’-5’) | target fragment (bp) |
| --- | --- | --- | --- |
| Rw10J19 | GCGAGTTGACGACGAGTT | GGGTGGGCTTCCTTAGTTA | 373 |
| Rw3N19 | CTGGCTGGTTCTCTTTCTG | ATGGGTCGTCGTCGATATG | 125 |
| Rw5D11 | CAGATTCGCCGTAGCCCTTAC | ATCCGAACCCCGACCTGAC | 254 |
| Rw11E5 | GATACCGCGAAGGTGTAGT | GAGTGAAAACTCTGCAATCA | 173 |
| Rw23H5 | AAGCTCTGCCATTGTCCACT | GCCCCTCCAAACTTAACCTC | 125 |
| Rw27A11B | TGTTCCCTTTTAATGAATTAGC | GTTCATCCCTTCAAACCAC | 313 |
| Rw46O8 | ACATGTGGTGCTGTGTTT | GTACAGGCCACTGCTGTC | 307 |
| Rw48N6 | GAGGGCGATCTTCGTATTCTC | GGGGCAATTGAAGGGTTTAG | 272 |
| Rw50N23 | AATTGGTATATTCTGGTAAGTAG | ATAGGAGGGTGATGACTAAC | 567 |
| Rw55C6 | GTGGATTTTCAGAGATACGC | TCACAGACAGGACCACCTAT | 265 |
| Rw61F2 | GTTGGAATTGCAGAGGTGAT | AACTAAAGGCAGGCCACTAA | 250 |
| Rog5 | ATTTTAGTTTCCTAGAGCAGA | ATCGTGGTATTTCATCGG | 141-153 |
| Rog9 | TCCTGAAAACGAAGCCTCC | TTCTCCGCCCTATCCAATG | 136-152 |
| Rog26 | CCGCACCCTACCTAAAAT | ATTGAATGGTTGAGATGCC | 123-141 |
| Rog27 | CTGGTGGAATATTTCATGC | TGGAATACAGAATCAAGAACA | 201-221 |
| C172 | ACAACACCAACTAGAACTTGAGC | GCTCAACAGCAACAACCTCA | 138-158 |
| C187 | TGTGCCTCGAGAGGTTTCTT | GTCAGCTGAAGCACTGGTGA | 197-209 |
| H9B01 | TCTGGTTGGTGATGATGAGC | CGTAAACCATTCCGTGTTCC | 232 |
| H10D03 | CAATTCAAAACCACCGCTCT | CGCAGAGTCAACGAACCATA | 222 |
| H20D08 | TTCGGCTCTCTTCTCTGCTC | GACATTACAGCGACGAAGCA | 240 |
| H22E04 | GACATCACCACCACCACAAG | AACCAAGGTTTCCAGTTCCA | 241 |
